# Supplementary material for: The Acute VertEbRal AugmentaTion (AVERT) study: protocol for a randomised controlled, feasibility trial of spinal medial branch nerve block in hospitalised older patients with vertebral fragility fractures
Source: BMJ Open. 2022 Jun 12;12(6):e059194. doi: 10.1136/bmjopen-2021-059194 (PMC9196181; doi:10.1136/bmjopen-2021-059194)
Supplement: Supplementary data [file bmjopen-2021-059194supp001.pdf]

AVERT Acute VertEbRal augmentaTion study

Nottingham University Hospitals NHS Trust

Participant Consent Form (Patient)

The AVERT (Acute Vertebral Fracture Augmentation) Study

Chief Investigator: Professor Opinder Sahota

Patient Screening No.: **SN** ☐☐☐ Initials: ☐☐☐

Patient initial each box

1. I confirm that I have read and understood the information sheet dated (/ /) (Version ___ for the above study and have had the opportunity to ask questions. ☐
2. I understand that my participation is voluntary and that I am free to withdraw at any time without my medical care or legal rights being affected. ☐
3. I understand that I may be invited to take part in a qualitative interview and that written quotations from the interview, that will be anonymized, may be used in publications, reports and presentations ☐
4. I understand that my medical records and any other information collected for the study may be looked at by authorised individuals from the Sponsor for the study, the funding body, and the UK Regulatory Authority in order to check that the study is being carried out correctly. ☐
5. I understand that even if I withdraw or am withdrawn from the above named study, that the data already collected from me will be retained and used when analysing the results of the study. ☐
6. I consent to the secure storage including electronic, of personal information for the purposes of this study. I understand that any information that could identify me will be kept strictly confidential and that no personal information will be included in the study report, presentations or other publication. ☐
7. I agree to undergo, where required, the tests and procedures as described in the information sheet. ☐
8. I agree that my GP, or any other doctor treating me, will be notified of my participation in this study. ☐
9. I agree to take part in the study ☐

Name of the patient *(Print)*	date	Patient's signature
Name of person taking consent *(Print)*	date	Signature

Original to be retained and filed in the site file. 1 copy to patient, 1 copy to be filed in patient's notes.

Tan CW, *et al. BMJ Open* 2022; 12:e059194. doi: 10.1136/bmjopen-2021-059194
