# Supplementary material for: The Acute VertEbRal AugmentaTion (AVERT) study: protocol for a randomised controlled, feasibility trial of spinal medial branch nerve block in hospitalised older patients with vertebral fragility fractures
Source: BMJ Open. 2022 Jun 12;12(6):e059194. doi: 10.1136/bmjopen-2021-059194 (PMC9196181; doi:10.1136/bmjopen-2021-059194)
Supplement: Author's manuscript [file bmjopen-2021-059194.draft_revisions.pdf]

BMJ Open

BMJ Open is committed to open peer review. As part of this commitment we make the peer review history of every article we publish publicly available.

When an article is published we post the peer reviewers' comments and the authors' responses online. We also post the versions of the paper that were used during peer review. These are the versions that the peer review comments apply to.

The versions of the paper that follow are the versions that were submitted during the peer review process. They are not the versions of record or the final published versions. They should not be cited or distributed as the published version of this manuscript.

BMJ Open is an open access journal and the full, final, typeset and author-corrected version of record of the manuscript is available on our site with no access controls, subscription charges or pay-per-view fees (http://bmjopen.bmj.com).

If you have any questions on BMJ Open's open peer review process please email info.bmjopen@bmj.com

BMJ Open

The Acute VertEbRal AugmentaTion (AVERT) Study: A randomised controlled, feasibility trial: Trial Protocol

Journal:	*BMJ Open*
Manuscript ID	bmjopen-2021-059194
Article Type:	Protocol
Date Submitted by the Author:	11-Nov-2021
Complete List of Authors:	Tan, Chia Wei; Nottingham University Hospitals NHS Trust, Health Care of the Older People<br>Cameron, Maribel; Nottingham University Hospitals NHS Trust, HCOP<br>Arlachov, Yuriy; Nottingham University Hospitals NHS Trust, Department of Radiology<br>Bastounis, Anastasios; University of Nottingham, Division of Epidemiology & Public Health<br>Bishop, Simon; Nottingham University, Nottingham University Business School<br>Czernicki, Michal; Nottingham University Hospitals NHS Trust, Department of Anaesthesia<br>Drummond, Avril; University of Nottingham, School of Health Sciences<br>Fakis, Apostolos; Royal Derby Hospital, Derby Clinical Trials Support Unit<br>Pasku, Dritan; Nottingham University Hospitals NHS Trust, Centre for Spinal Studies and Surgery<br>Sahota, Opinder; Nottingham University Hospitals NHS Trust, HCOP
Keywords:	Pain management < ANAESTHETICS, GERIATRIC MEDICINE, Bone diseases < ORTHOPAEDIC & TRAUMA SURGERY, Spine < ORTHOPAEDIC & TRAUMA SURGERY

SCHOLARONE™
Manuscripts

TITLE PAGE:

The Acute VertEbRal AugmentaTion (AVERT) Study: A randomised controlled, feasibility trial: Trial Protocol

Author: Chia Wei Tan

Health Care of Older People Division, South Block, B floor, Queens Medical Centre, Debry Road, Nottingham, UK, NG7 2UH

Telephone: 0115 924 9924. Ext 82323

Correspondence To: Chia Wei Tan, chia.tan@nuh.nhs.uk

Co- Authors:

1. **Maribel Cameron**: Healthcare of the Older People Division, Nottingham University Hospitals NHS Trust, Nottingham, UK
2. **Yuriy Arlachov:** Department of Radiology, Nottingham University Hospitals NHS Trust, Nottingham, UK
3. **Anastasios Bastounis:** Division of Epidemiology & Public Health, University of Nottingham, Nottingham, UK
4. **Simon Bishop:** Nottingham University Business School, University of Nottingham, Nottingham, UK
5. **Michal Czernicki**: Department of Anaesthesia, Nottingham University Hospitals NHS Trust, Nottingham, UK
6. **Avril Drummond**: School of Health Sciences, University of Nottingham, Nottingham, UK
7. **Apostolos Fakis**: Derby Clinical Trials Support Unit, University Hospitals of Derby and Burton NHS Foundation Trust, Derby, UK
8. **Dritan Pasku**: Centre for Spinal Studies and Surgery, Nottingham University Hospitals NHS Trust, Nottingham, UK
9. **Opinder Sahota**:
   Healthcare of the Older People Division, Nottingham University Hospitals NHS Trust, Nottingham, UK
   Centre for Spinal Studies and Surgery, Nottingham University Hospitals NHS Trust, Nottingham, UK
   Division of Rehabilitation, Ageing and Wellbeing, University of Nottingham, Nottingham, UK
   National Institute for Health Research (NIHR) Nottingham Biomedical Research Centre (BRC), Nottingham, UK

Keywords:
Vertebral fractures, osteoporosis, vertebroplasty, nerve-block, randomised controlled trial

Word Count: 4560 excluding abstract, figures, tables and references.

For peer review only

ABSTRACT

Introduction

Vertebral Fragility Fractures (VFF) are the most common type of osteoporotic fracture found in older people, resulting in increasing morbidity and excess mortality. These fractures can cause significant pain, requiring admission to hospital. Vertebroplasty (VP) is effective in reducing pain and allowing early mobilisation in hospitalised patients. However, it may be associated with complications such as cement leakage, infection, bleeding at the injection site and fracture of adjacent vertebrae. It is also costly and not readily accessible in many UK hospitals.

A recent retrospective study reported that spinal medial branch nerve block (MBNB), typically used to treat facet arthropathy, had similar efficacy in terms of pain relief compared to VP for the treatment of painful VFF. However, to date no study has prospectively compared MBNB to VP. We therefore propose a prospective feasibility RCT to compare the role of MBBM to VP, in hospitalised older patients.

Method

A parallel, two-arm randomised controlled trial with participants allocated on a 1:1 ratio to either standard care-VP or MBNB in hospitalised patients aged over 70. Follow up will be at weeks 1, 4 and 8 post intervention. The primary objective is to determine the feasibility and design of a future trial, including specific outcomes of recruitment, adherence to randomisation and safety. Embedded within the trial will be a health economic evaluation to understand resource utilisation and implications of the intervention and a qualitative study of the experiences and insights of trial participants and clinicians. Secondary outcomes will include pain scores, analgesia requirements, resource use and quality of life data

Discussion

VP is recommended for hospitalised patients with acute pain not responding to medical management, however is expensive and associated with complications. By comparison, MBNB is less invasive, can be delivered by non-specialist units and, if effective, could lead to a change in clinical practice.

Ethics and Dissemination

Ethical Approval was granted by the Yorkshire & the Humber Research Ethics Committee (**reference 21/YH/0065**). AVERT has received approval by the Health Research Authority (**reference IRAS 293210**) and is sponsored by Nottingham University Hospitals NHS Trust (**reference 21HC001**). Recruitment is ongoing. Results will be presented at relevant conferences and submitted to appropriate journals for publication on completion.

Trial Registration Number

ISRCTN 18334053 ; Registered on 27/5/2021. Pre-recruitment and Pre-results

For peer review only

Article Summary

Strengths and Limitations of this Study

- The two interventions are proven, safe, surgical interventions, already used in existing healthcare practice and adverse events from both interventions are already well documented.
- The descriptive analysis on effectiveness of outcome measures will inform hypothesis testing in a future definitive trial, including levels of variability required to determine power.
- It will determine the feasibility of collecting economic measures, including detailed resource use and quality of life data within the two arms, which will aid the design of a comprehensive economic evaluation in a future definitive trial.
- The nested semi-structured qualitative interviews will provide valuable data to inform future trial acceptability for participants and the health care professionals involved in their clinical care.

INTRODUCTION

Vertebral fragility fractures (VFF) are the most common osteoporotic fracture, leading to both acute and chronic back pain, substantial spinal deformity, functional disability, decreased quality of life and increased mortality[1, 2]. The presence of one VFF increases the risk of a new VFF five-fold, and up to twelve-fold in the presence of two or more VFF[3,4].These fractures frequently occur with very little trauma from day-to-day activities, such as bending forward, twisting, lifting light objects, and sitting from a standing position onto a low chair[5].

Patients with VFF may have mild to moderate symptoms, however a significant proportion require hospitalisation due to disabling pain[6]. The incidence ranges from 10 to 20 per 10,000/year, rising to 50 per 10,000/year in those aged 80 years and over[7]. Hospitalised VFF patients tend to be older, frail with coexisting comorbidities and have cognitive impairment. These patients have longer hospital stay and higher mortality[7, 8]. Conservative (non-surgical) treatment for these patients consists of bed-rest, analgesia and, controversially, thoracolumbar bracing[9]. These are poorly tolerated, and immobilisation leads to muscle wasting, impaired rehabilitation, further bone loss and medical complications such as chest infections, deep venous thrombosis and pulmonary[10]. Potent morphine-based analgesia leads to nausea, constipation and particularly in those with cognitive impairment and delirium[11]. Moreover, 1 in 5 patients are re-admitted within 30 days[12].

Vertebroplasty (VP) is a minimally invasive, image-guided key-hole procedure that involves injection of radio-opaque bone cement into the fractured vertebral body, in an effort to provide pain relief and stability[13]. Recent guidelines recommend VP as the first line treatment for VFF, given its efficacy, cost and minimally invasive nature compared to Balloon Kyphoplasty (BKP), with BKP reserved more traumatic fractures in younger people[14]. Although a Cochrane review concluded that VP had no important benefits[16], the limitations of the review were recognised [17, 18]. VP performed within 3 weeks of fracture in older hospitalised patients with severe pain has been recommended [19, 20], demonstrating a

For peer review only

reduction in pain, pain related disability and hospital length of stay compared to conservative medical treatment [12, 21-23]. A reduction in hospital and long term mortality has also been reported when compared with conservative treatment [24-27].

With ageing demographics, we anticipate an increasing demand for VP in hospitalised patients nationally. From our local audit data, 81 patients had VP in Nottingham last year. This amounted to a cost of £650,000, with a single level VP costing £4,500. The literature reports complications (range 2.2% - 3.9%) related to VP, which includes cement leakage, infection, bleeding at the injection site, fracture of the ribs, posterior elements or pedicle[27].

Recent studies have shown that steroid injections at the facet joint to block the medical branches from the dorsal rami spinal nerve roots – medial branch nerve block (MBNB)- may be effective in managing pain in patients with VFF [29]. A retrospective study demonstrated that MBNB had similar efficacy in terms of pain relief and radiological changes after 2 years follow up [30], however its application in older hospitalised patients needs to be explored. We hypothesize that MBNB will be as effective clinically and cost effective compared to VP, will be more readily accessible via anaesthetic and interventional radiology service, and will eliminate the risks of adjacent vertebral fracture and cement leakage associated with VP.

METHODS AND ANALYSIS

Aims

The ultimate aim of the feasibility trial is to prepare the foundations to design and conduct a multi-centre, randomised controlled trial of hospitalised older people with painful VFF in order to evaluate the clinical and cost effectiveness of MBNB compared to routine surgical treatment.

Objectives

The feasibility of conducting a definitive randomised controlled trial will be determined by considering the following objectives:

- Determine the number of patients who meet the eligibility criteria in addition to the recruitment (including willingness to be randomised) and retention rates of those eligible patients;
- Test several outcome measures for assessment of mobility, pain and quality of life, for their potential use as a primary outcome measure.
- Calculate means and standard deviations for the quantitative measures to allow hypothesis testing and development of the analysis plan for a future definitive trial;
- Evaluate ease of access and availability of information from primary and secondary care databases to determine the most efficient way of measuring patient level healthcare costs.
- Use of a qualitative nested interview study to assess participants' and clinicians' views on trial acceptability, trial processes and define the 'non-inferiority' margin to inform the design and conduct of a future definitive trial.

For peer review only - http://bmjopen.bmj.com/site/about/guidelines.xhtml

Study Design

The study design is a parallel, two-arm randomised controlled feasibility trial with participants individually randomised on a 1:1 ratio to continue with their planned surgical care-VP or MBNB treatment. Embedded within the feasibility study will be a health economic analysis to understand resource utilisation and implications of the intervention and a qualitative study which will focus on the experiences of participants and clinicians involved in the study, their insights and recommendations for improving trial acceptability and processes.

Participants

This is single site study (Queens Medical Centre, Nottingham University Hospitals NHS trust) with a catchment population of 800,000. Participants presenting with acute painful VFF and awaiting spinal surgery will be approached for recruitment into the study.

The inclusion and exclusion criteria are:

Inclusion criteria

- Patients aged 70 years and over admitted to hospital;
- Ambulatory prior to injury;
- < 6 weeks from date of injury;
- Numeric Rating Scale pain score 7 or more on standing;
- MRI confirmed oedema at the site of fracture;

Exclusion criteria

- Debilitating chronic back pain not relieved despite opiate use;
- Substantial fracture retropulsion; acute infection or spinal malignancy;
- 3 or more acute vertebral fractures
- Receiving palliative care
- Spinal deformity which contraindicates VP

For peer review only

STUDY Procedures

Recruitment

Patients who fulfil the eligibility criteria will be invited to participate. Those who indicate an interest will be introduced to the research team and details of the study will be explained: a patient information sheet (PIS) will be provided. Patients will have 24 hours to consider participation before providing informed consent. Consent will be obtained in accordance with Good Clinical Practice guidance, and will include consent for potential inclusion in the qualitative interview study. **Figure 1** illustrates the process of obtaining consent for the study.

An Abbreviated Mental Test (AMT) on admission for potential participants will be used as a screening tool for capacity assessment. A patient is deemed to have capacity if their AMT is ≥7/10 at either point of assessment. If AMT is documented as <7/10 then it will be repeated by the research team at the time of screening, and prompt a capacity assessment in relation to research.

For Potential participants who are unable to provide consent, relatives or carers will be approached in the capacity of the participants' personal consultee.

Patients who decline to take part, or consultees who decline the patient's participation in the study, will be asked if they would be willing to share their reasons. The information will be valuable for us to improve future trial design and acceptability. However there will be no requirement to do so and it will not affect the care that their relative/friend receives in hospital if they decline to share this information. The findings will be tabulated into the final results.

Randomisation

Participants will be randomly allocated to routine care VP or MBNB. Allocation (Block randomisation) will be in a 1:1 ratio via a secure web based system (RedCap Cloud). VP and MBNB will be undertaken within 72 hours of randomisation where possible. Participants and their GPs will be notified of their allocated arm in the study and a record of randomisation will be made in the participant's medical notes.

Due to the nature of the study, it will not be possible to blind the participant or staff to VP surgery or MBNB.

Intervention

Intervention Group

This will be performed by a consultant anaesthetist or an interventional radiologist trained in MBNB

Bilateral Medial Branch Nerve Blocks (MBNB) will be performed targeting facet joints above and below the vertebral fracture. Contrast media, oblique and lateral XR views will be used

For peer review only

to assess optimal position of the needle. A mixture of 0.5 % Bupivacaine with 40 mg Depomedrone will be used. Each medial branch will be blocked with 1-1.5 mls solution.

Standard care Group

VP. Usual post-operative care and monitoring will follow after the surgery. Participants will be encouraged to mobilise as pain allows and be prescribed analgesia as required.

Outcomes

Feasibility Study outcomes:

- Number of eligible patients
- Rate of participant recruitment and randomisation
- Reasons why participants are not recruited or randomised
- Rate of participant adherence to randomisation (cross-over) and retention
- Completion of study rates and reasons for non-completion
- Completeness of data
- Standard deviation and effect size of potential outcomes for subsequent definitive trial
- Time from randomisation to delivery of the intervention

Assessment of outcome measures used in feasibility study for its potential use in a future definitive trial

- Functional disability as measured by the 24 point Roland Morris Disability Questionnaire (RMDQ)[30] (This is the most commonly used outcome measure in previous studies)
- Pain as measured by the 0-10 Numeric Pain Rating Scale (NRS-11) [31]
- Quality of Life as measured by the EuroQol (EQ5D-5L) and where appropriate, proxy EQ5D-5L [32]
- Activities of daily living as measured by the Nottingham Extended Activities of Daily Living (NEADL) scale [33]
- Abbreviated Mental Test (AMT) for cognition assessment [34]
- Montreal Cognitive Assessment for cognition assessment [35]
- Charlson Co-morbidity index as a predictive tool for 1-year mortality [36]
- Clinical Frailty Scale (CFS)– frailty assessment [37]
- Sociodemography data including age, sex, socio-economic status
- Record of pain medication use (using the opioid dose equivalence table)

Analgesia Requirement

Analgesia requirement will be recorded as follows: each medication will be classified as a strong opioid (including oxycodone, morphine, fentanyl, pethidine, hydromorphone, buprenorphine and tramadol), mild opioid (including medications containing codeine or dextropropoxyphene) or non-opioid medication (including paracetamol and non-steroidal anti-inflammatory drugs (NSAIDs)). The participant will be given a score of 0, 1 or 2 in each of these three categories depending on the number of concurrent different medications being

For peer review only

taken within each category. Opioid medication will also include a calculation of the oral Morphine Equivalent Daily Dose using the Opioid Dose Equivalence score[38].

Follow up

Participant flow through the study is summarised in **Figure 2**. Follow up timings will be counted from the time of intervention. Participants will be followed up face to face at week 1 (±3 days) (whilst in hospital), week 4 (±7 days, telephone interviews) and week 8 (±7 days, telephone interviews).

Follow up outcome measures will include:

- Participant still living (established by the hospital's NHS spine portal enquiry).
- Hospital length-of-stay (ascertained by the hospital electronic database, supplemented by review of medical notes by a different member of staff to maintain blinding, if necessary)
- Unplanned hospital re-admission within 28 and 91 days post discharge (ascertained by the hospital electronic database, supplemented by review of medical notes if necessary).
- Health economics/resource utilisation (patient and NHS costs)
- Time from randomisation to delivery of the intervention.
- Quality of life measures using RMDQ[30],EQ-5D-5L[32]
- NEADL[33]
- NRS[31]

Qualitative assessments

The study will be complemented by a nested semi-structured interview to provide essential insights into the feasibility, design and conduct of a definitive trial. This will focus on the experiences of participants (n=10) and clinicians (n=5) in the study, their insights and their recommendations for improving trial acceptability and processes.

A range of participants with different characteristics which we believe might lead them to have a different opinion or experience will be invited for the interview. For example, participants of both genders; participants from both arms of the study, a spectrum of participants on the clinical frailty scale; some patients who may have particular communication needs (eg deafness, English as a second language speakers). Personal consultees of participants will also be invited to participate in the interview. Similarly, we wish to explore clinical staffs' thoughts about participant recruitment (eligibility and randomisation), an acceptable non-inferiority margin, as well as reflect upon the process of integrating the research with the clinical service. Clinical staff will be identified by the study lead, who will distribute an invitation letter/email and information sheet to eligible staff. All clinicians will be provided with a participant information leaflet, and asked to give written, informed consent, using a dedicated clinician interview consent form.

All interviews will be audio recorded, transcribed in full and anonymised using a professional transcription service.

For peer review only

Sample Size Calculation

Approximately 200 patients present with acute VFF each year (local audit data) of which approximately 1/3 require VP. We propose to recruit for 10 months, in which time we expect to approach approximately 50 patients listed for VP. Assuming 80% are eligible, and with a 70% consent rate, we expect to recruit 30 participants. Allowing for a 10% two month attrition rate, 28 participants will complete the study. By recruiting 30 patients we will be able to estimate a recruitment rate of 82% (CI 74%, 92%) and a retention rate of 90% (CI 81%, 99%). Completed follow-up on 28 participants will allow the Roland Morris Disability mean score to be estimated, with an approximate standard error of 0.98, assuming a SD of 6, thus enabling future power calculations to be calculated. Sample sizes between 24 and 40 are recommended for a feasibility study [39-41] and thus by recruiting 30 participants, we are confident we can determine the feasibility of conducting a definitive trial.

Data Analysis

A statistical analysis plan will be finalised and approved prior to database lock and commencement of data analysis. Feasibility outcomes will be summarised using appropriate descriptive statistics; mean (95% Confidence Interval) for continuous and frequency (%) for categorical. Completeness and descriptive summaries of outcome data at each follow up time point will be presented. Descriptive summaries of NHS costs (using standard unit costs) data at each follow up time point will be presented.

Economic Evaluation

In addition, at week 8, to inform the definitive economic analysis, we will assess resource use between VP and MBNB treatments; the ease of access to information about resource use from routine database systems; and the feasibility of collecting such data.

Surgical VP and MBNB treatment resource use will be collected from the medical notes and through discussions with participants by the research staff. The cost associated with the surgery will be based on the recorded resource use for the surgery (eg. Consumables, equipment, grade and number of nursing staff present during the operation). Further health resource information will be extracted from the hospital electronic system (NotIS) and the GP electronic systems by the research assistant. This will include any outpatient appointment, outpatient procedures, emergency department visits, inpatient admissions related to the study or GP visits captured at baseline, week 4 and week 8.

The unit costs of health resources will be based on national tariffs such as the Unit Costs of Health and Social Care for primary care resources, NHS Reference Costs for secondary care resources and the BNF for prescriptions. These tariffs will be estimated at 2021 prices. We will also utilise national databases such as the NHS Healthcare Resource Group (HRG) Tariff [42], the Personal Social Services Research Unit (PSSRU) Costs of Health [43], the Office of National Statistics (ONS) Bulletin Annual Survey of Hours and Earnings[44] and NHS Reference Costs (https://www.gov.uk/government/collections/nhs-reference-costs). Any unit cost that is not available will be estimated in consultation with the hospital finance department.

For peer review only

Health Economic Analysis

We will rehearse the cost-effective analysis to inform the study hypothesis and the analysis plan for the definitive trial. The within-trial economic evaluation will determine the cost and outcome of VP and MBNB treatment from a NHS perspective. The evaluation will follow the reference case guidance for technology appraisals as set out by NICE[45] .Effectiveness will be captured using quality adjusted life years (QALYs) as assessed by the EQ-5D-5L[32] at 8 weeks. The primary health economic outcome of the evaluation will be the incremental cost per additional quality-adjusted life years (QALY) gained from surgical VP and MBNB. To control for the impact of uncertainty, one- and two-way sensitivity analyses will be performed on (but not exclusively) age, gender and baseline scores. The impact of parameter uncertainty will also be addressed using a probabilistic sensitivity analysis, allowing the calculation of 95% confidence intervals for the incremental cost-effectiveness ratio and cost-effectiveness acceptability curves.

Qualitative analysis

Data will handled using the NVivo software package and analysed using a framework thematic approach. The framework will be informed by the literature around the challenges of clinical trial methodology[46-51], and initial themes are likely to include elements such as randomisation, outcome measures, communication and feedback. Data from each interview will be mapped to the thematic tables.

Harms

The intervention is not testing a new surgical technique or medical treatment, and VP and MBNB are recognised as safe and routinely used procedures, hence AEs will be recorded in the CRFs, but will not require expedited reporting to the sponsor or REC.

All AEs will be reviewed by the chief investigator (CI) and recorded as part of the study outcome measures with an assessment of the severity, relation and expectation. All deaths occurring up to the final study visit and serious AEs, other than expected surgical complications, will be recorded on the Sponsor SAE form and emailed to the Sponsor within 3 days of a researcher becoming aware of the event. Those related to the study and unexpected will be reported to the REC within 15 days. Events will be followed up until resolved or a final outcome has been reached.

ETHICS and DISSEMNATION

Patient and Public involvement

The study's Patient and Public Involvement (PPI) group are members of the Royal Osteoporosis Society's Nottingham Support group. The topic is well recognized amongst the PPI who highlighted this area for further research. The member's experiences have greatly influenced the design of the research study and the choice of proposed outcome measures of the study. They will be able to comment on aspects of the study design and contribute to production of questionnaire booklets, information sheets and other documents to ensure these are understandable and acceptable to patients. They will also be invited to comment on methods of sharing the study findings and support the writing of the definitive, future trial application.

For peer review only

Dissemination Policy

The team's dissemination strategy aims to target relevant policy makers and patient groups through our PPI representative. We will publish the study protocol and the full report of AVERT will be available on the NIHR RfPB website. Results from the trial will also be submitted for presentation at scientific meetings and conferences targeted at clinicians working with older people, trauma and spinal surgery.

Study Registration and Approvals

AVERT has received approval from the Research Ethics Committee (REC—Yorkshire & The Humber – Bradford Leeds, reference number 21/YH/0065), Health Research Authority (HRA) and the Nottingham Queens Medical Centre Research & Innovation department. Nottingham University Hospitals NHS Trust will act as sponsor to this study. The study has been registered on a clinical trials database (https://www.isrctn.com, reference number ISRCTN 18334053, pre-results).

DISCUSSION

Across Europe, 22 million women and 5 million men have osteoporosis, with 4 million new fragility fractures occurring a year, at an estimated health and social care cost of £37 billion [52] which is predicted to double by 2050 [53]. One in 2 women and 1 in 5 men over the age of 50 will experience and osteoporotic fracture in their lifetime, with this rising by 25% over the next 5 years [54]. In the UK, around 3.5 million people have osteoporosis, with an annual incidence of 500,000 of new fragility fractures [55]. Vertebral fragility fractures (VFF) are the most common osteoporotic fracture. Prevalence studies suggest 20% of women aged over 80 years have sustained a VFF [56].

The majority of those with pain symptoms will have mild to moderate pain, however a significant proportion have severe pain and require admission to hospital. The annual incidence of hospitalisation with symptomatic VFF is rising (50 per 10,000/year in those aged 80 years and over) [5], and these figures could be still be underestimated as up to 70% of vertebral fractures remain undiagnosed [5, 57]. In one study, it was reported that 34% of patients with VFF required acute hospital admission [58], with an average length of stay of 15 days in the UK [59]. Hospitalised VFF patients tend to be older, frail with coexisting comorbidities, cognitive impairment and have worse physical-health-related quality of life [60]. With the increasing numbers of VFF and more patients requiring acute hospitalisation, the healthcare resource burden within this group of patients is alarming.

Current NICE guidance on management of VFF emphasises a trial of pain optimisation before surgical intervention (vertebral augmentation) is considered. Potent morphine based analgesia, which are frequently required, lead to nausea, constipation and particularly in those with cognitive impairment, delirium [11]. Vertebral augmentation is a general term for several techniques used to treat painful VFF, with the aim of consolidating the fracture and, when possible, achieve height restoration. Vertebroplasty (VP) is a minimally invasive, image-guided key-hole procedure that involves injection of radio-opaque bone cement into the fractured vertebral body, in an effort to provide pain relief and stability [13]. Percutaneous balloon kyphoplasty (PBK) attempts to restore vertebral body height by inflating a balloon

For peer review only

prior to bone cement injection [14]. Although the recent Cochrane review has debated the effectiveness of VP [15, 16, 17], limitations in the review are recognised [16, 17]. Emerging evidence suggests that the optimal benefits are seen when VP is performed within 3 weeks of fracture in hospitalised older patients [19, 20]

Debate has been generated regarding the effectiveness of VP and its application in patients with vertebral fractures following a Cochrane review. However, the data is limited for VP and its application for inpatients [18, 19], even less so for older hospitalized patients. The VAPOUR trial, which had a subgroup of inpatients (59%) with vertebral fractures, demonstrated that vertebroplasty showed benefit in terms of pain reduction mortality, length of stay in hospital [19]. The other trials (VERTOS4 [61], Kalllmes et al [62], Buchbinder et al [63]) highlighted in the Cochrane review had no inpatients and recruited from an outpatient setting. As far as we are aware, the AVERT trial will be the first trial to focus exclusively on hospitalised older people.

It is also worth noting that previous trials did not include patients who had a diagnosis of dementia or delirium during recruitment. As cognitively impaired older patients presenting to hospital with VFF account for a large proportion of the real world VFF cohort [64], it would severely affect the validity and generalisability of the trial to exclude them. However, we are unaware of any published RCT on VP that have included patients with cognitive impairment. We believe that this cohort of patients would benefit most from early intervention for their painful VFF as they may struggle to comply with regular analgesia intake, and opiate based analgesia which is usually required may have subsequent side effects that will precipitate delirium itself.

Additionally, our participants will receive treatment for their painful VFF with either VP, a NICE approved treatment for VFF, as our control group, or our trial intervention with MBNB. Should MBNB prove as effective as VP in controlling pain, it also negates the need for a general anaesthetic for this population group, which is viewed as potentially more harmful in the frail, older population. In this trial, we also propose that surgical management should be considered earlier in the treatment of hospitalisation , improving pain with the aim of preventing pain-related immobilisation and disability, therefore reducing risks of opiate induced side effects and consequences of prolonged immobilisation. The timely intervention (within 72 hours of randomisation) we propose in this feasibility trial will be dependent on other factors such as timely identification of acute VFF, MRI imaging availability, operator and theatre availability, where more emergent procedures will take precedence. This is in addition to the already growing emergency list from the impact of the COVID-19 pandemic [65, 66].

The alternative solution to this is to devise a new clinical pathway for these patients. Hence it is essential that the feasibility trial is delivered within the framework of the existing healthcare service in order to facilitate implementation. As we may be potentially expanding clinical workload in other departments (ie interventional radiology and anaesthesia) should MBNB prove effective, the feasibility of this must be explored and limitations and constraints in an already pressured healthcare system. Cost effectiveness of MBNB must also be explored compared to standard care VP, hence the inclusion of an economic evaluation. A benefit of a new clinical service would be that MBNB could be delivered in local hospitals via anaesthetic or interventional radiology departments. This facilitates early treatment for painful VFF and eliminates the need for our patients to travel to regional spine centres for VP. This sentiment

For peer review only

was echoed in a UK study which highlighted concerns regarding access to VP nationally for our older hospitalised patients with VFF [28].

The key outcomes address questions posed by the possible limitations of conducting such a trial within an existing public health service, specifically to recruitment and adherence to randomisation. We anticipate that this feasibility study will provide information and data to test our hypothesis that MBNB is as cost effective and safe compared to VP for the treatment of acute VFF in hospitalised older patients. With national organisational leads pushing for better outcomes for frail older patients [67, 68], we hope that this trial will be a further catalyst for change nationally in the management of older hospitalised patients with VFF.

Trial Status

The trial is currently open for recruitment, with 12 patients currently enrolled (November 2021).
We aim to complete the study in September 2022.

Author affiliations:

1. Healthcare of the Older People Division, Nottingham University Hospitals NHS Trust, Nottingham, UK
2. Department of Radiology, Nottingham University Hospitals NHS Trust, Nottingham, UK
3. Division of Epidemiology & Public Health, University of Nottingham, Nottingham UK
4. Nottingham University Business School, University of Nottingham, Nottingham, UK
5. Department of Anaesthesia, Nottingham University Hospitals NHS Trust, Nottingham, UK
6. School of Health Sciences, University of Nottingham, Nottingham, UK
7. Derby Clinical Trials Support Unit, University Hospitals of Derby and Burton NHS Foundation Trust
8. Centre for Spinal Studies and Surgery, Nottingham University Hospitals NHS Trust, Nottingham, UK
9. Division of Rehabilitation, Ageing and Wellbeing, University of Nottingham, Nottingham, UK
10. National Institute for Health Research (NIHR) Nottingham Biomedical Research Centre (BRC), Nottingham, UK

Acknowledgements

We would like to express our sincere thanks and gratitude to our PPI representative (**LW**) for her contribution to the original protocol and the NIHR RfPB for funding this study.

Author Contributors

For peer review only

CWT wrote the manuscript. **YA, AB, SB, MC, AD, NP, LW and OS** are all key protocol contributors providing their expertise on specific aspects of the study. **OS** is the chief investigator of the study. **MC** is the trial co-ordinator. **AF** is the lead trial statistician. All authors have read, contributed amendments to and approved the final manuscript.

Funding

This paper presents independent research funded by the National Institute for Health Research (NIHR) under its Research for Patient Benefit (RfPB) Programme. (Grant Reference number **NIHR RfPB-201937**)

Disclaimer

Views and opinions expressed in this publication are those of the authors.

Neither sponsor nor funder had or will have direct input in the study conduct, data analysis, data interpretation, and manuscript writing and dissemination findings.

Competing Interests

None Declared.

Patient consent for publication.

Not Required.

Provenance and peer review

Not commissioned; peer-reviewed for ethical and funding approval prior to submission.

For peer review only

REFERENCES

1. Silverman SL, Minshall ME, Shen W, et al., Health-Related Quality of Life Subgroup of the Multiple Outcomes of Raloxifene Evaluation Study. The relationship of health-related quality of life to prevalent and incident vertebral fractures in postmenopausal women with osteoporosis: results from the Multiple Outcomes of Raloxifene Evaluation Study. Arthritis Rheum 2001;44:2611–19.
2. Cohen LD. Fractures of the osteoporotic spine. Orthop Clin N Am 1990;21:143–50.
3. Melton LJ 3rd, Atkinson EJ, Cooper C, O'Fallon WM,Riggs BL. Vertebral fractures predict subsequent fractures. Osteoporosis Int. 1999;10:214-21.
4. Black DM, Arden NK, Palermo L, Pearson J, Cummings SR. Prevalent vertebral deformities predict hip fractures and new vertebral deformities but not wrist fractures. Study of Osteoporotic Fractures Research Group. J Bone Miner Res 1999;14:821-28.
5. Cooper C1, Atkinson EJ, Jacobsen SJ, O'Fallon WM, Melton LJ 3rd. Population-based study of survival after osteoporotic fractures. Am J Epidemiol 1993 May;137:1001-5.
6. Jacobsen SJ, Cooper C, Gottlieb MS, Goldberg J, Yahnke DP, Melton LJ. Hospitalization with vertebral fracture among the aged: a national population-based study, 1986–1989. Epidemiology 1992; 3: 515–18.
7. Ong T, Kantachuvesiri P, Sahota O, Gladman JRF. Characteristics and outcomes of hospitalised patients with vertebral fragility fractures: a systematic review. Age Ageing. 2018 Jan 1;47(1):17-25.
8. Walters S, Chan S, Goh L, Ong T, Sahota O. The Prevalence of Frailty in Patients Admitted to Hospital with Vertebral Fragility Fractures. Curr Rheumatol Rev 2016;12:244-247.
9. Abe T, Shibao Y, Takeuchi Y, Mataki Y, Amano K, Hioki S, Miura K, Noguchi H, Funayama T, Koda M, Yamazaki M. Initial hospitalization with rigorous bed rest followed by bracing and rehabilitation as an option of conservative treatment for osteoporotic vertebral fractures in elderly patients: a pilot one arm safety and feasibility study. Arch Osteoporos 2018; 23:134-138.
10. Goldstein CL, Chutkan NB, Choma TJ, Orr RD. Management of the elderly with vertebral compression fractures. Neurosurgery 2015; 77:33–45.
11. Chau DL, Walker V, Pai L, Cho LM. Opiates and elderly: Use and side effects Clin Interv Aging 2008; 3:273–278.
12. Tsai YW, Hsiao FY, Wen YW, et al. Clinical outcomes of vertebroplasty or kyphoplasty for patients with vertebral compression fractures: a nationwide cohort study. J Am Med Dir 2013;14:41–47
13. Lieberman IH, Togawa D, Kayanja MM. Vertebroplasty and kyphoplasty: filler materials. Spine J. 2005;5:S305–16.
14. Robinson Y, Heyde CE, Fo¨rsth P, Olerud C. Kyphoplasty in osteoporotic vertebral compression fractures—guidelines and technical considerations. J Orthop Surg Res 2011;6:43-47.
15. Tsoumakidou G, CW, Koch G, et al. CIRSE Guidelines on Percutaneous Vertebral Augmentation. Cardiovasc Intervent Radiol 2017; 40:331–342.
16. Cochrane Database of Systematic Reviews. Percutaneous vertebroplasty for osteoporotic vertebral compression fracture. Cochrane Systematic Review - Intervention Version published: 06 November 2018; https://doi.org/10.1002/14651858.CD006349.pub4.
17. Clark W, Bird P, Diamond T et al. EBM Analysis General medicine, Cochrane vertebroplasty review misrepresented evidence for vertebroplasty with early intervention in severely affected patients http://dx.doi.org/10.1136/bmjebm-2019-11117.
18. https://www.cochranelibrary.com/cca/doi/10.1002/cca.2376/full .
19. Diamond T, Clark W, Bird P et al. Early vertebroplasty within 3 weeks of fracture for acute painful vertebral osteoporotic fractures: subgroup analysis of the VAPOUR trial and review of the literature.
    European Spine Journal. 2020: https://doi.org/10.1007/s00586-020-06362-2.

20. Corteta B, Chastanet P, Laredo J-D. Plea for continuing but rational use of vertebroplasty for osteoporotic vertebral fractures. Joint Bone Spine 2020;87:281–283.
21. Zhu RS, Kan SL, Ning GZ, Chen LX, Cao ZG, Jiang ZH, Zhang XL, Hu W. Which is the best treatment of osteoporotic vertebral compression fractures: balloon kyphoplasty, percutaneous vertebroplasty, or non-surgical treatment? A Bayesian network meta-analysis. Osteoporos Int 2019;30:287-298.
22. Zhang L, Zhai P. A Comparison of Percutaneous Vertebroplasty Versus Conservative Treatment in Terms of Treatment Effect for Osteoporotic Vertebral Compression Fractures: A Meta-Analysis. Surg Innov 2020;27:19-25.
23. Maravic M, Taupin P, Roux C. Hospital burden of vertebral fractures in France: influence of vertebroplasty. Osteoporosis Int 2013;24:2001–06.
24. Edidin AA, Ong KL, Lau E, et al. Morbidity and mortality after vertebral fractures: comparison of vertebral augmentation and nonoperative management in the Medicare population. Spine 2015;40:1228–41.
25. Lange A, Kasperk C, Alvares L, et al. Survival and cost comparison of kyphoplasty and percutaneous vertebroplasty using German claims data. Spine 2014;39:318–26.
26. Lin JH, Chien LN, Tsai WL, et al. Early vertebroplasty associated with a lower risk of mortality and respiratory failure in aged patients with painful vertebral compression fractures: a population-based cohort study in Taiwan. Spine J 2017;17:1310–18.
27. Saracen A, Kotwica Z. Complications of percutaneous vertebroplasty: an analysis of 1100 procedures performed in 616 patients. Medicine (Baltimore). 2016;95:e3850.
28. Ong T, Gladman JRF, Sahota O. Management of Patients Admitted of Hospital with Vertebral Fractures-A Modified Delphi Study. Age and Ageing. 2020: In press
29. Mitra R, Do H, Alamin T, Cheng I. Facet pain in thoracic compression fractures. Pain Med 2010;11:1674-1677
30. Bae IS, Chun HJ, Bak KH, Yi HJ, Choi KS, Kim KD. Medial Branch Block Versus Vertebroplasty for 1-Level Osteoporotic Vertebral Compression Fracture: 2-Year Retrospective Study. World Neurosurg. 2019 Feb;122:e1599-e1605.
31. Roland MO, Morris RW. A study of the natural history of back pain. Part 1: Development of a reliable and sensitive measure of disability in low back pain. Spine 1983; 8: 141-144.
32. McCaffery M, Pasero C. Pain: Clinical Manual, St. Louis, 1999, P. 16. Copyrighted by Mosby, Inc.
33. http://www.euroqol.org/eq-5d-products/eq-5d-5l.html.
34. Green J and Young J. A test-retest reliability study of the Barthel Index, the Rivermead Mobility Index, the Nottingham extended Activities of Daily Living Scale and the Frenchay Activities Index. Journal Disability and Rehabilitation 2001; 23: 234-39.
35. Hodkinson HM. Evaluation of a mental test score for assessment of mental impairment in the elderly. Age Ageing. 1972;1(4):233-8.
36. Nasreddine ZS, Phillips NA, Bédirian V, et al. The Montreal Cognitive Assessment, MoCA: a brief screening tool for mild cognitive impairment. J Am Geriatr Soc. 2005;53(4):695–9.
37. Charlson ME, Pompei P, Ales KL, MacKenzie CR. A new method of classifying prognostic comorbidity in longitudinal studies: development and validation. J Chronic Dis 1987;40(5):373-383.
38. Rockwood K, Song X, MacKnight C, et al. A global clinical measure of fitness and frailty in elderly people. CMAJ. 2005;173(5):489–95.
39. Faculty of Pain Medicine. Opioid Dose Equivalence: calculation of oral Morphine Equivalent Daily Dose (oMEDD). Australian and New Zealand College of Anaesthetists. Available at: http://www.fpm.anzca.edu.au/resources/professional-documents/ OPIOID%20DOSE%20EQUIVALENCE.pdf. Date accessed March 2015.

For peer review only

40. Watson J, Torgerson D. Increasing recruitment to randomised trials: a review of randomised controlled trials. BMC Med Res Methodol 2006; 6: 34-38.
41. Treweek S, Pitkethly M, Kjeldstrom M, Taskila T, Johansen M, Sullivan F et al. Strategies to improve recruitment to randomised clinical trials (Review). Cochrane Database SystRev 2010; Issue 4: No. MR000013.
42. Browne RH. On the use of a pilot sample for sample size determination. Stat Med. 1995; 14:1933–1940.
43. https://improvement.nhs.uk/resources/national-tariff-2021-consultation/
44. https://www.pssru.ac.uk/project-pages/unit-costs/unit-costs-2019/
45. https://www.ons.gov.uk/surveys/informationforbusinesses/businesssurveys/annualsurveyofhoursandearningsashe
46. National Institute for Health and Care Excellence. NICE Technology Appraisal Guidance. https://www. nice. org. uk/ about/ what- we- do/ our- programmes/ nice-guidance/ nice- technology- appraisal- guidance (Accessed May 2019).
47. Watson J, Torgerson D. Increasing recruitment to randomised trials: a review of randomised controlled trials. BMC Med Res Methodol 2006; 6: 34-38.
48. Treweek S, Pitkethly M, Kjeldstrom M, Taskila T, Johansen M, Sullivan F et al. Strategies to improve recruitment to randomised clinical trials (Review). Cochrane Database SystRev 2010; Issue 4: No. MR000013.
49. Browne RH. On the use of a pilot sample for sample size determination. Stat Med. 1995; 14:1933–1940.
50. Lancaster GA, Dodd S, Williamson PR. Design and analysis of pilot studies: recommendations for good practice. J Eval Clin Practice 2004;10:307-312
51. Sim J, Lewis M. The size of a pilot study for a clinical trial should be calculated in relation to considerations of precision and efficiency. J Clin Epidemiol 2012;65:301-308.
52. Hernlund E, Svedbom A, Ivergård M, Compston J, Cooper C, Stenmark J, McCloskey EV, Jönsson B, Kanis JA. Osteoporosis in the European Union: medical management, epidemiology and economic burden. A report prepared in collaboration with the International Osteoporosis Foundation (IOF) and the European Federation of Pharmaceutical Industry Associations (EFPIA). Arch Osteoporos 2013;8:136-142.
53. Reginster JY, Burlet N. Osteoporosis: A still increasing prevalence. Bone. 2006 Feb;38 (2 Suppl 1):S4-9.
54. Borgström F, Zethraeus N, Johnell O, et al. Costs and quality of life associated with osteoporosis related fractures in Sweden. Osteoporos Int 2006;17:637–50.
55. BROKEN BONES, BROKEN LIVES: A roadmap to solve the fragility fracture crisis in the United Kingdom.
56. O'Neill TW, Felsenberg D, Varlow J, Cooper C, Kanis JA, Silman J. The prevalence of vertebral deformity in European men and women: the European Vertebral Osteoporosis Study. J Bone Miner Res. 1996;11:1010-18.
57. Alendronate, etidronate, risedronate, raloxifene, strontium ranelate and teriparatide for the secondary prevention of osteoporotic fragility fractures in postmenopausal women (TA161). NICE: 2008. Available from: https://www.nice. org.uk/guidance/ta161 [Last updated 9 Aug. 2017].
58. Johansen A et al. Trauma admissions in the elderly how does a patient's age affect the likelihood of their being admitted to hospital after a fracture? Injury Vol 29, No 10.
59. Osteoporosis in the European Union 2008. Ten years of Progress and ongoing challenges. International Osteoporosis foundation.
60. Al-Sari U, Tobias J, Clark E. Health related quality of life in older people with osteoporotic vertebral fractures: a systematic review and meta-analysis. Osteoporosis Int. 2016;27(10),2891-909.

For peer review only

61. Firanescu C, Lohle PN, de Vries J, et al. VERTOS IV study group. Arandomised sham controlled trial of vertebroplasty for painful acute osteoporotic vertebral fractures (VERTOS IV). Trials 1745;2011:12–93.
62. Kallmes DF, Comstock BA, Heagerty PJ, et al. A randomized trial of vertebroplasty for osteoporotic spinal fractures. New England Journal of Medicine 2009;361:569–79.
63. Buchbinder R, Osborne RH, Ebeling PR, et al. A randomized trial of vertebroplasty for painful osteoporotic vertebral fractures. N Engl J Med2009;361:557–68.
64. Ong T, Sahota O, Gladman JRF. The Nottingham Spinal Health (NoSH) Study: a cohort study of patients hospitalised with vertebral fragility fractures. Osteoporosis International 2020; 31:363–370
65. Too long to wait: the impact of COVID-19 on elective surgery Lancet Rheumatology, ISSN: 2665-9913, Vol: 3, Issue: 2, Page: e83; 2021.
66. Covid-19: Waiting times in England reach record highsBMJ 2020; 370 doi: https://doi.org/10.1136/bmj.m3557
67. https://www.scfn.org.uk/SpecialisedClinicalFrailtyToolkitVersion4 - December 2019
68. https://www.gettingitrightfirsttime.co.uk/spinal-surgery-report/

For peer review only

Figure 1. Flowchart Guidance for Consent

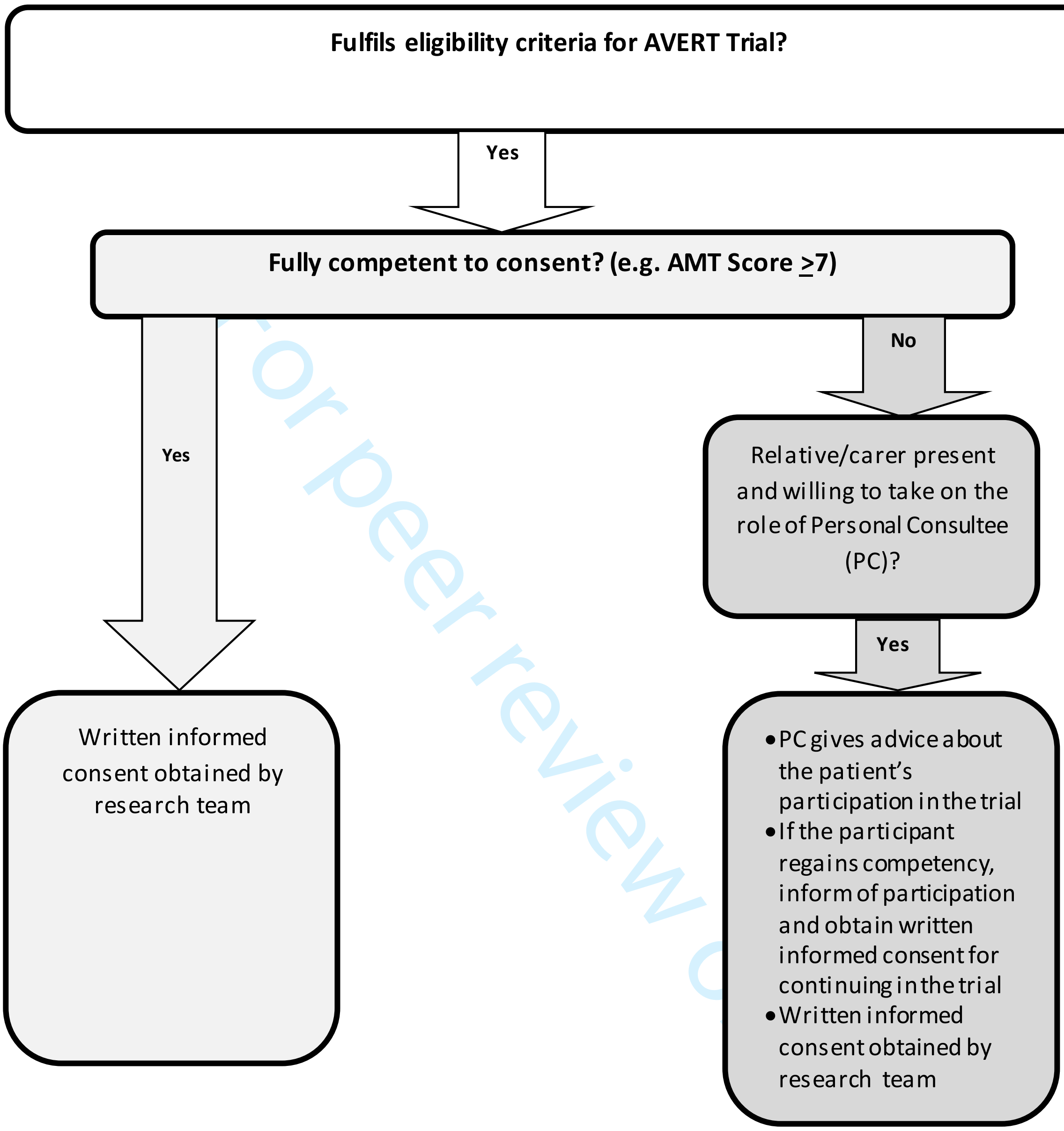

If a participant loses capacity during the study period, they will continue in the study unless there is a known change in their views/perspectives determined by liaising with their relative/carer.

For participants who initially lack capacity but later regain capacity, eg with delirium we will re-approach them, detailing their participation in the study to date, provide a patient information sheet and discuss the purpose and nature of the research, its benefits (or lack of), risks and burdens; understand the alternatives to taking part. If they agree to continue with participation in the study, we will request to sign and updated consent form. If they decline they will be categorised as withdrawn.

For peer review only

Figure 2. TRIAL FLOW CHART

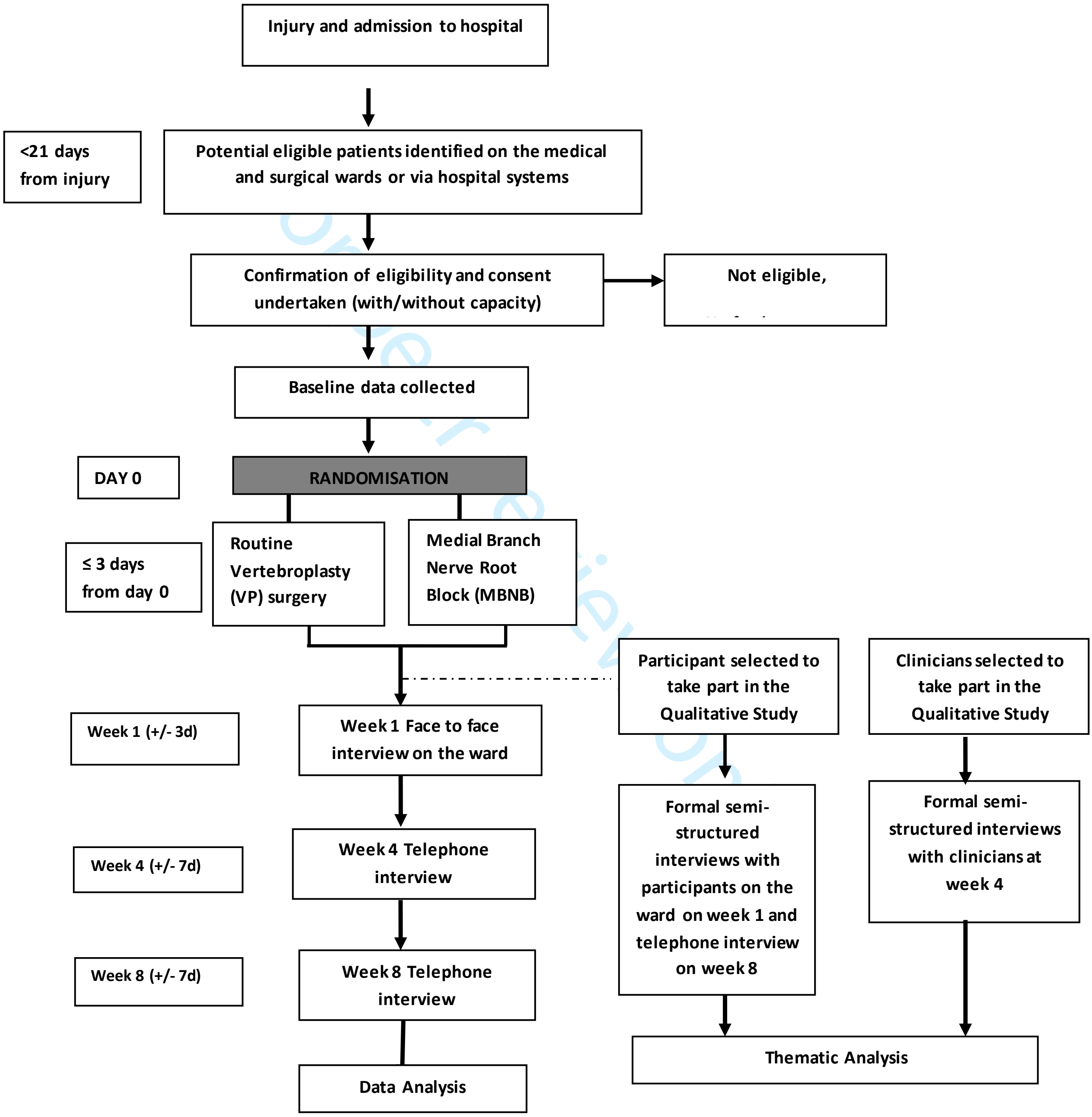

For peer review only

Appendix 1 – Schedule of Procedures

Procedures	Screening	Baseline	Day of surgery	Week 1 Face-to-face	Week 4 Telephone	Week 8 Telephone
Visit Window	<21 days from injury	Day 0	≤ 3 Days from Randomisation	+/-3 days (or post intervention)	+/- 7 days	+/- 7 days
Eligibility Assessment	x	x	x	x	X	x
Abbreviated Mental Test (AMT)*	x*			x		x[2]
Participant and fracture details	x	x				
Informed consent		x				
Randomisation		x				
Surgery details & complications			x[1]	x[1]	x[1]	x[1]
Charlson Co-morbidity Assessment		x				
Clinical Frailty Scale (CFS)		x				
Roland Morris Disability Questionnaire				x	x	x
NEADL		x		x	x	x

For peer review only

**Montreal – Cognitive Assessment (MoCA)++**		x				
**Numeric Pain Rating Scale++**		x		x	x	x
**EQ-5D-5L++**		x		x	x	x
**Analgesia requirement**		x		x	x	x
**Healthcare resource utilisation ****		x	x	x	x	x
**Qualitative study interviews**				x[2]	x[3]	
**Participant safety review**			x	x	x	x

[1] Participants in the surgery intervention arm. [2] 10 participants. [3] 5 participants

* AMT will be assessed at the point of hospital admission as part of routine care..

** Healthcare resource utilisation will be captured at discharge and throughout the study.
All participant questionnaires will be completed where possible, however for those participants that lack capacity, these will not be obtainable, just the clinically assessed ones.

For peer review only

SPIRIT
STANDARD PROTOCOL ITEMS: RECOMMENDATIONS FOR INTERVENTIONAL TRIALS

SPIRIT 2013 Checklist: Recommended items to address in a clinical trial protocol and related documents*

Section/item	Item No	Description
**Administrative information**		
Title (Done)	1	Descriptive title identifying the study design, population, interventions, and, if applicable, trial acronym
Trial registration (Done)	2a	Trial identifier and registry name. If not yet registered, name of intended registry
	2b	All items from the World Health Organization Trial Registration Data Set
Protocol version (Done)	3	Date and version identifier
Funding (Done)	4	Sources and types of financial, material, and other support
Roles and responsibilities (Done)	5a	Names, affiliations, and roles of protocol contributors
	5b	Name and contact information for the trial sponsor
	5c	Role of study sponsor and funders, if any, in study design; collection, management, analysis, and interpretation of data; writing of the report; and the decision to submit the report for publication, including whether they will have ultimate authority over any of these activities
	5d	Composition, roles, and responsibilities of the coordinating centre, steering committee, endpoint adjudication committee, data management team, and other individuals or groups overseeing the trial, if applicable (see Item 21a for data monitoring committee)
**Introduction**		
Background and rationale (Done)	6a	Description of research question and justification for undertaking the trial, including summary of relevant studies (published and unpublished) examining benefits and harms for each intervention
	6b	Explanation for choice of comparators
Objectives (Done)	7	Specific objectives or hypotheses

For peer review only

Trial design (Done)	8	Description of trial design including type of trial (eg, parallel group, crossover, factorial, single group), allocation ratio, and framework (eg, superiority, equivalence, noninferiority, exploratory)

Methods: Participants, interventions, and outcomes

Study setting (Done)	9	Description of study settings (eg, community clinic, academic hospital) and list of countries where data will be collected. Reference to where list of study sites can be obtained
Eligibility criteria (Done)	10	Inclusion and exclusion criteria for participants. If applicable, eligibility criteria for study centres and individuals who will perform the interventions (eg, surgeons, psychotherapists)
Interventions (Done)	11a	Interventions for each group with sufficient detail to allow replication, including how and when they will be administered
	11b	Criteria for discontinuing or modifying allocated interventions for a given trial participant (eg, drug dose change in response to harms, participant request, or improving/worsening disease)
	11c	Strategies to improve adherence to intervention protocols, and any procedures for monitoring adherence (eg, drug tablet return, laboratory tests)
	11d	Relevant concomitant care and interventions that are permitted or prohibited during the trial
Outcomes (Done)	12	Primary, secondary, and other outcomes, including the specific measurement variable (eg, systolic blood pressure), analysis metric (eg, change from baseline, final value, time to event), method of aggregation (eg, median, proportion), and time point for each outcome. Explanation of the clinical relevance of chosen efficacy and harm outcomes is strongly recommended
Participant timeline (Done)	13	Time schedule of enrolment, interventions (including any run-ins and washouts), assessments, and visits for participants. A schematic diagram is highly recommended (see Figure)
Sample size (Done)	14	Estimated number of participants needed to achieve study objectives and how it was determined, including clinical and statistical assumptions supporting any sample size calculations
Recruitment (Done)	15	Strategies for achieving adequate participant enrolment to reach target sample size

Methods: Assignment of interventions (for controlled trials)

Allocation:

Sequence generation (Done)	16a	Method of generating the allocation sequence (eg, computer-generated random numbers), and list of any factors for stratification. To reduce predictability of a random sequence, details of any planned restriction (eg, blocking) should be provided in a separate document that is unavailable to those who enrol participants or assign interventions
Allocation concealment mechanism (Done)	16b	Mechanism of implementing the allocation sequence (eg, central telephone; sequentially numbered, opaque, sealed envelopes), describing any steps to conceal the sequence until interventions are assigned
Implementation (Done)	16c	Who will generate the allocation sequence, who will enrol participants, and who will assign participants to interventions
Blinding (masking) (Done)	17a	Who will be blinded after assignment to interventions (eg, trial participants, care providers, outcome assessors, data analysts), and how
	17b	If blinded, circumstances under which unblinding is permissible, and procedure for revealing a participant's allocated intervention during the trial
**Methods: Data collection, management, and analysis**		
Data collection methods (Done)	18a	Plans for assessment and collection of outcome, baseline, and other trial data, including any related processes to promote data quality (eg, duplicate measurements, training of assessors) and a description of study instruments (eg, questionnaires, laboratory tests) along with their reliability and validity, if known. Reference to where data collection forms can be found, if not in the protocol
	18b	Plans to promote participant retention and complete follow-up, including list of any outcome data to be collected for participants who discontinue or deviate from intervention protocols
Data management (Done)	19	Plans for data entry, coding, security, and storage, including any related processes to promote data quality (eg, double data entry; range checks for data values). Reference to where details of data management procedures can be found, if not in the protocol
Statistical methods (Done)	20a	Statistical methods for analysing primary and secondary outcomes. Reference to where other details of the statistical analysis plan can be found, if not in the protocol
	20b	Methods for any additional analyses (eg, subgroup and adjusted analyses)
	20c	Definition of analysis population relating to protocol non-adherence (eg, as randomised analysis), and any statistical methods to handle missing data (eg, multiple imputation)

For peer review only - http://bmjopen.bmj.com/site/about/guidelines.xhtml

Methods: Monitoring

Data monitoring (Done)	21a	Composition of data monitoring committee (DMC); summary of its role and reporting structure; statement of whether it is independent from the sponsor and competing interests; and reference to where further details about its charter can be found, if not in the protocol. Alternatively, an explanation of why a DMC is not needed
	21b	Description of any interim analyses and stopping guidelines, including who will have access to these interim results and make the final decision to terminate the trial
Harms (Done)	22	Plans for collecting, assessing, reporting, and managing solicited and spontaneously reported adverse events and other unintended effects of trial interventions or trial conduct
Auditing (Done)	23	Frequency and procedures for auditing trial conduct, if any, and whether the process will be independent from investigators and the sponsor

Ethics and dissemination

Research ethics approval (Done)	24	Plans for seeking research ethics committee/institutional review board (REC/IRB) approval
Protocol amendments (Done)	25	Plans for communicating important protocol modifications (eg, changes to eligibility criteria, outcomes, analyses) to relevant parties (eg, investigators, REC/IRBs, trial participants, trial registries, journals, regulators)
Consent or assent (Done)	26 a	Who will obtain informed consent or assent from potential trial participants or authorised surrogates, and how (see Item 32)
	26b	Additional consent provisions for collection and use of participant data and biological specimens in ancillary studies, if applicable
Confidentiality (Done)	27	How personal information about potential and enrolled participants will be collected, shared, and maintained in order to protect confidentiality before, during, and after the trial
Declaration of interests (Done)	28	Financial and other competing interests for principal investigators for the overall trial and each study site
Access to data (Done)	29	Statement of who will have access to the final trial dataset, and disclosure of contractual agreements that limit such access for investigators
Ancillary and post-trial care (Done)	30	Provisions, if any, for ancillary and post-trial care, and for compensation to those who suffer harm from trial participation

For peer review only

Dissemination policy (Done)	31a	Plans for investigators and sponsor to communicate trial results to participants, healthcare professionals, the public, and other relevant groups (eg, via publication, reporting in results databases, or other data sharing arrangements), including any publication restrictions
	31b	Authorship eligibility guidelines and any intended use of professional writers
	31c	Plans, if any, for granting public access to the full protocol, participant-level dataset, and statistical code
**Appendices**		
Informed consent materials (Supplied if necessary)	32	Model consent form and other related documentation given to participants and authorised surrogates
Biological specimens (N/A)	33	Plans for collection, laboratory evaluation, and storage of biological specimens for genetic or molecular analysis in the current trial and for future use in ancillary studies, if applicable

*It is strongly recommended that this checklist be read in conjunction with the SPIRIT 2013 Explanation & Elaboration for important clarification on the items. Amendments to the protocol should be tracked and dated. The SPIRIT checklist is copyrighted by the SPIRIT Group under the Creative Commons "Attribution-NonCommercial-NoDerivs 3.0 Unported" license.

For peer review only

For peer review only - http://bmjopen.bmj.com/site/about/guidelines.xhtml

BMJ Open

The Acute VertEbRal AugmentaTion (AVERT) Study: Protocol for a randomised controlled, feasibility trial of spinal medial branch nerve block in hospitalised older patients with vertebral fragility fractures

Journal:	*BMJ Open*
Manuscript ID	bmjopen-2021-059194.R1
Article Type:	Protocol
Date Submitted by the Author:	28-Mar-2022

Complete List of Authors:	Tan, Chia Wei; Nottingham University Hospitals NHS Trust, Health Care of the Older People<br>Cameron, Maribel; Nottingham University Hospitals NHS Trust, HCOP<br>Arlachov, Yuriy; Nottingham University Hospitals NHS Trust, Department of Radiology<br>Bastounis, Anastasios; University of Nottingham, Division of Epidemiology & Public Health<br>Bishop, Simon; Nottingham University, Nottingham University Business School<br>Czernicki, Michal; Nottingham University Hospitals NHS Trust, Department of Anaesthesia<br>Drummond, Avril; University of Nottingham, School of Health Sciences<br>Fakis, Apostolos; Royal Derby Hospital, Derby Clinical Trials Support Unit<br>Pasku, Dritan; Nottingham University Hospitals NHS Trust, Centre for Spinal Studies and Surgery<br>Sahota, Opinder; Nottingham University Hospitals NHS Trust, HCOP

**Primary Subject Heading**:	Geriatric medicine
Secondary Subject Heading:	Surgery
Keywords:	Pain management < ANAESTHETICS, GERIATRIC MEDICINE, Bone diseases < ORTHOPAEDIC & TRAUMA SURGERY, Spine < ORTHOPAEDIC & TRAUMA SURGERY

SCHOLARONE™
Manuscripts

TITLE PAGE:

The Acute VertEbRal AugmentaTion (AVERT) Study: Protocol for a randomised controlled, feasibility trial of spinal medial branch nerve block in hospitalised older patients with vertebral fragility fractures

Author: Chia Wei Tan

Health Care of Older People Division, South Block, B floor, Queens Medical Centre, Debry Road, Nottingham, UK, NG7 2UH

Telephone: 0115 924 9924. Ext 82323

Correspondence To: Chia Wei Tan, cwtan@doctors.org.uk

Co- Authors:

1. **Maribel Cameron**: Healthcare of the Older People Division, Nottingham University Hospitals NHS Trust, Nottingham, UK
2. **Yuriy Arlachov:** Department of Radiology, Nottingham University Hospitals NHS Trust, Nottingham, UK
3. **Anastasios Bastounis:** Division of Epidemiology & Public Health, University of Nottingham, Nottingham, UK
4. **Simon Bishop:** Nottingham University Business School, University of Nottingham, Nottingham, UK
5. **Michal Czernicki**: Department of Anaesthesia, Nottingham University Hospitals NHS Trust, Nottingham, UK
6. **Avril Drummond**: School of Health Sciences, University of Nottingham, Nottingham, UK
7. **Apostolos Fakis**: Derby Clinical Trials Support Unit, University Hospitals of Derby and Burton NHS Foundation Trust, Derby, UK
8. **Dritan Pasku**: Centre for Spinal Studies and Surgery, Nottingham University Hospitals NHS Trust, Nottingham, UK
9. **Opinder Sahota**:
   Healthcare of the Older People Division, Nottingham University Hospitals NHS Trust, Nottingham, UK
   Centre for Spinal Studies and Surgery, Nottingham University Hospitals NHS Trust, Nottingham, UK
   Division of Rehabilitation, Ageing and Wellbeing, University of Nottingham, Nottingham, UK
   National Institute for Health Research (NIHR) Nottingham Biomedical Research Centre (BRC), Nottingham, UK

Keywords:
Vertebral fractures, osteoporosis, vertebroplasty, nerve-block, randomised controlled trial

Word Count: 4675 excluding abstract, figures, tables and references.

For peer review only

ABSTRACT

Introduction

Vertebral Fragility Fractures (VFF) are the most common type of osteoporotic fracture found in older people, resulting in increasing morbidity and excess mortality. These fractures can cause significant pain, requiring admission to hospital. Vertebroplasty (VP) is effective in reducing pain and allowing early mobilisation in hospitalised patients. However, it may be associated with complications such as cement leakage, infection, bleeding at the injection site and fracture of adjacent vertebrae. It is also costly and not readily accessible in many UK hospitals.

A recent retrospective study reported that spinal medial branch nerve block (MBNB), typically used to treat facet arthropathy, had similar efficacy in terms of pain relief compared to VP for the treatment of painful VFF. However, to date no study has prospectively compared MBNB to VP. We therefore propose a prospective feasibility RCT to compare the role of MBNB to VP, in hospitalised older patients.

Method

A parallel, two-arm randomised controlled trial with participants allocated on a 1:1 ratio to either standard care-VP or MBNB in hospitalised patients aged over 70 with acute osteoporotic vertebral fractures. Follow up will be at weeks 1, 4 and 8 post intervention. The primary objective is to determine the feasibility and design of a future trial, including specific outcomes of recruitment, adherence to randomisation and safety. Embedded within the trial will be a health economic evaluation to understand resource utilisation and implications of the intervention and a qualitative study of the experiences and insights of trial participants and clinicians. Secondary outcomes will include pain scores, analgesia requirements, resource use and quality of life data.

Ethics and Dissemination

Ethical Approval was granted by the Yorkshire & the Humber Research Ethics Committee (**reference 21/YH/0065**). AVERT has received approval by the Health Research Authority (**reference IRAS 293210**) and is sponsored by Nottingham University Hospitals NHS Trust (**reference 21HC001**). Recruitment is ongoing. Results will be presented at relevant conferences and submitted to appropriate journals for publication on completion.

Trial Registration Number

ISRCTN 18334053 ; Registered on 27/5/2021. Pre-recruitment and Pre-results

For peer review only

Article Summary

Strengths and Limitations of this Study

- The two interventions are proven, safe, surgical interventions, already used in existing healthcare practice and adverse events from both interventions are already well documented.
- The descriptive analysis on effectiveness of outcome measures will inform hypothesis testing in a future definitive trial, including levels of variability required to determine power.
- It will determine the feasibility of collecting economic measures, including detailed resource use and quality of life data within the two arms, which will aid the design of a comprehensive economic evaluation in a future definitive trial.
- The nested semi-structured qualitative interviews will provide valuable data to inform future trial acceptability for participants and the health care professionals involved in their clinical care.

INTRODUCTION

Vertebral fragility fractures (VFF) are the most common osteoporotic fracture, leading to both acute and chronic back pain, substantial spinal deformity, functional disability, decreased quality of life and increased mortality[1, 2]. The presence of one VFF increases the risk of a new VFF five-fold, and up to twelve-fold in the presence of two or more VFF[3,4].These fractures frequently occur with very little trauma from day-to-day activities, such as bending forward, twisting, lifting light objects, and sitting from a standing position onto a low chair[5].

Patients with VFF may have mild to moderate symptoms, however a significant proportion require hospitalisation due to disabling pain[6]. The incidence ranges from 10 to 20 per 10,000/year, rising to 50 per 10,000/year in those aged 80 years and over[7]. Hospitalised VFF patients tend to be older, frail with coexisting comorbidities and have cognitive impairment. These patients have longer hospital stay and higher mortality[7, 8]. Conservative (non-surgical) treatment for these patients consists of bed-rest, analgesia and, controversially, thoracolumbar bracing[9]. These are poorly tolerated, and immobilisation leads to muscle wasting, impaired rehabilitation, further bone loss and medical complications such as chest infections, deep venous thrombosis and pulmonary embolism[10]. Potent morphine-based analgesia leads to nausea, constipation and particularly in those with cognitive impairment and delirium[11]. Moreover, 1 in 5 patients are re-admitted within 30 days[12].

Vertebroplasty (VP) is a minimally invasive, image-guided key-hole procedure that involves injection of radio-opaque bone cement into the fractured vertebral body, in an effort to provide pain relief and stability[13]. Recent guidelines recommend VP as the first line treatment for VFF, given its efficacy, cost and minimally invasive nature compared to Balloon Kyphoplasty (BKP), with BKP reserved for more traumatic fractures in younger people[14]. Although a Cochrane review concluded that VP had no important benefits[15], the limitations of the review were recognised [16, 17]. VP performed within 3 weeks of fracture in older hospitalised patients with severe pain has been recommended [18, 19], demonstrating a reduction in pain, pain related

For peer review only

disability and hospital length of stay compared to conservative medical treatment [12, 20-22]. A reduction in hospital and long term mortality has also been reported when compared with conservative treatment [23-26].

With ageing demographics, we anticipate an increasing demand for VP in hospitalised patients nationally. From our local audit data, 81 patients had VP in Nottingham last year. This amounted to a cost of £650,000, with a single level VP costing £4,500. The literature reports complications (range 2.2% - 3.9%) related to VP, which includes cement leakage, infection, bleeding at the injection site, fracture of the ribs, posterior elements or pedicle[26].

Recent studies have shown that steroid injections at the facet joint to block the medical branches from the dorsal rami spinal nerve roots – medial branch nerve block (MBNB)- may be effective in managing pain in patients with VFF [27]. A retrospective study demonstrated that MBNB had similar efficacy in terms of pain relief and radiological changes after 2 years follow up [28], however its application in older hospitalised patients needs to be explored. We hypothesize that MBNB will be as effective clinically and cost effective compared to VP, will be more readily accessible via anaesthetic and interventional radiology service, and will eliminate the risks of adjacent vertebral fracture and cement leakage associated with VP.

METHODS AND ANALYSIS

Aims

The ultimate aim of the feasibility trial is to prepare the foundations to design and conduct a multi-centre, randomised controlled trial of hospitalised older people with painful VFF in order to evaluate the clinical and cost effectiveness of MBNB compared to routine surgical treatment.

Objectives

The feasibility of conducting a definitive randomised controlled trial will be determined by considering the following objectives:

- Determine the number of patients who meet the eligibility criteria in addition to the recruitment (including willingness to be randomised) and retention rates of those eligible patients;
- Test several outcome measures for assessment of mobility, pain and quality of life, for their potential use as a primary outcome measure.
- Calculate means and standard deviations for the quantitative measures to allow hypothesis testing and development of the analysis plan for a future definitive trial;
- Evaluate ease of access and availability of information from primary and secondary care databases to determine the most efficient way of measuring patient level healthcare costs.
- Use of a qualitative nested interview study to assess participants' and clinicians' views on trial acceptability, trial processes and define the 'non-inferiority' margin to inform the design and conduct of a future definitive trial.

For peer review only

Study Design

The study design is a parallel, two-arm randomised controlled feasibility trial with participants individually randomised on a 1:1 ratio to continue with their planned surgical care-VP or MBNB treatment. Embedded within the feasibility study will be a health economic analysis to understand resource utilisation and implications of the intervention and a qualitative study which will focus on the experiences of participants and clinicians involved in the study, their insights and recommendations for improving trial acceptability and processes.

Participants

This is single site study (Queens Medical Centre, Nottingham University Hospitals NHS trust) with a catchment population of 800,000. Participants presenting with acute painful VFF and awaiting spinal surgery will be approached for recruitment into the study.

The inclusion and exclusion criteria are:

Inclusion criteria

- Patients aged 70 years and over admitted to hospital;
- Ambulatory prior to injury;
- < 6 weeks from date of injury;
- Numeric Rating Scale pain score 7 or more on standing and not responding to analgesia;
- MRI confirmed oedema at the site of fracture;

Exclusion criteria

- Debilitating chronic back pain not relieved despite opiate use;
- Substantial fracture retropulsion; acute infection or spinal malignancy;
- 3 or more acute vertebral fractures
- Receiving palliative care
- Spinal deformity which contraindicates VP

For peer review only

STUDY Procedures

Recruitment

Patients who fulfil the eligibility criteria will be invited to participate. Those who indicate an interest will be introduced to the research team and details of the study will be explained: a patient information sheet (PIS) will be provided. Patients will have 24 hours to consider participation before providing informed consent. Consent (**Supplementary file**) will be obtained in accordance with Good Clinical Practice guidance, and will include consent for potential inclusion in the qualitative interview study. **Figure 1** illustrates the process of obtaining consent for the study.

An Abbreviated Mental Test (AMT) on admission for potential participants will be used as a screening tool for capacity assessment. A patient is deemed to have capacity if their AMT is ≥7/10 at either point of assessment. If AMT is documented as <7/10 then it will be repeated by the research team at the time of screening, and prompt a capacity assessment in relation to research.

For Potential participants who are unable to provide consent, relatives or carers will be approached in the capacity of the participants' personal consultee.

Patients who decline to take part, or consultees who decline the patient's participation in the study, will be asked if they would be willing to share their reasons. The information will be valuable for us to improve future trial design and acceptability. However there will be no requirement to do so and it will not affect the care that their relative/friend receives in hospital if they decline to share this information. The findings will be tabulated into the final results.

Randomisation

Participants will be randomly allocated to routine care VP or MBNB. Allocation (Block randomisation) will be in a 1:1 ratio via a secure web based system (RedCap Cloud). VP and MBNB will be undertaken within 72 hours of randomisation where possible. Participants and their GPs will be notified of their allocated arm in the study and a record of randomisation will be made in the participant's medical notes.

Due to the nature of the study, it will not be possible to blind the participant or staff to VP surgery or MBNB.

Intervention

Intervention Group

This will be performed by a consultant anaesthetist or an interventional radiologist trained in MBNB.

Bilateral MBNB will be performed targeting facet joints above and below the vertebral fracture. Fluoroscopy will be used to assess the optimal position of the needle. A mixture of 0.5 % Bupivacaine with 40 mg Depomedrone will be used. Each medial branch will be blocked with 1-1.5 mls solution.

For peer review only

Standard care Group

VP

This will be performed by an interventional radiologist or spinal surgeon under general anaesthesia or conscious sedation using Fluoroscopy. The approach will be bipedicular or unipedicular, aiming the placement of the Jamshidi needle (8-12 G in size) at the centre of the vertebral body. A bony biopsy will always precede the injection of the Polymethylmethacrylate (PMMA) bone cement. Approximately, a volume of 2 ml to 5ml of the cement will be injected in the vertebral body according to the level and the morphology of the fracture, trying to minimize any leakage.

Usual post-operative care and monitoring will follow after the surgery. Participants will be encouraged to mobilise as pain allows and be prescribed analgesia as required.

For peer review only

Outcomes

Feasibility Study outcomes:

- Number of eligible patients
- Rate of participant recruitment and randomisation
- Reasons why participants are not recruited or randomised
- Rate of participant adherence to randomisation (cross-over) and retention
- Completion of study rates and reasons for non-completion
- Completeness of data
- Standard deviation and effect size of potential outcomes for subsequent definitive trial
- Time from randomisation to delivery of the intervention

Assessment of outcome measures used in feasibility study for its potential use in a future definitive trial

- Functional disability as measured by the 24 point Roland Morris Disability Questionnaire (RMDQ)[29] (This is the most commonly used outcome measure in previous studies)
- Pain as measured by the 0-10 Numeric Pain Rating Scale (NRS-11) [30]
- Quality of Life as measured by the EuroQol (EQ5D-5L) and where appropriate, proxy EQ5D-5L [31]
- Activities of daily living as measured by the Nottingham Extended Activities of Daily Living (NEADL) scale [32]
- Abbreviated Mental Test (AMT) for cognition assessment [33]
- Montreal Cognitive Assessment for cognition assessment [34]
- Charlson Co-morbidity index as a predictive tool for 1-year mortality [35]
- Clinical Frailty Scale (CFS)– frailty assessment [36]
- Sociodemography data including age, sex, socio-economic status
- Record of pain medication use (using the opioid dose equivalence table)

Analgesia Requirement

Analgesia requirement will be recorded as follows: each medication will be classified as a strong opioid (including oxycodone, morphine, fentanyl, pethidine, hydromorphone, buprenorphine and tramadol), mild opioid (including medications containing codeine or dextropropoxyphene) or non-opioid medication (including paracetamol and non-steroidal anti-inflammatory drugs (NSAIDs)). The participant will be given a score of 0, 1 or 2 in each of these three categories depending on the number of concurrent different medications being taken within each category. Opioid medication will also include a calculation of the oral Morphine Equivalent Daily Dose using the Opioid Dose Equivalence score[37].

Follow up

Participant flow through the study is summarised in **Figure 2**. Follow up timings will be counted from the time of intervention. Participants will be followed up face to face at week 1 (±3 days) (whilst in hospital), week 4 (±7 days, telephone interviews) and week 8 (±7 days, telephone interviews).

Follow up outcome measures will include:

- Participant still living (established by the hospital's NHS spine portal enquiry).
- Hospital length-of-stay (ascertained by the hospital electronic database, supplemented by review of medical notes by a different member of staff to maintain blinding, if necessary)
- Unplanned hospital re-admission within 28 and 91 days post discharge (ascertained by the hospital electronic database, supplemented by review of medical notes if necessary).
- Health economics/resource utilisation (patient and NHS costs)
- Time from randomisation to delivery of the intervention.
- Quality of life measures using RMDQ[29],EQ-5D-5L[31]
- NEADL[32]
- NRS[30]

Qualitative assessments

The study will be complemented by a nested semi-structured interview to provide essential insights into the feasibility, design and conduct of a definitive trial. This will focus on the experiences of participants (n=10) and clinicians (n=5) in the study, their insights and their recommendations for improving trial acceptability and processes.

A range of participants with different characteristics which we believe might lead them to have a different opinion or experience will be invited for the interview. For example, participants of both genders; participants from both arms of the study, a spectrum of participants on the clinical frailty scale; some patients who may have particular communication needs (eg deafness, English as a second language speakers). Personal consultees of participants will also be invited to participate in the interview. Similarly, we wish to explore clinical staffs' thoughts about participant recruitment (eligibility and randomisation), an acceptable non-inferiority margin, as well as reflect upon the process of integrating the research with the clinical service. Clinical staff will be identified by the study lead, who will distribute an invitation letter/email and information sheet to eligible staff. All clinicians will be provided with a

For peer review only

participant information leaflet, and asked to give written, informed consent, using a dedicated clinician interview consent form.

All interviews will be audio recorded, transcribed in full and anonymised using a professional transcription service.

Sample Size Calculation

Approximately 200 patients present with acute VFF each year (local audit data) of which approximately 1/3 require VP. We propose to recruit for 10 months, in which time we expect to approach approximately 50 patients listed for VP. Assuming 80% are eligible, and with a 70% consent rate, we expect to recruit 30 participants. Allowing for a 10% two month attrition rate, 28 participants will complete the study. By recruiting 30 patients we will be able to estimate a recruitment rate of 82% (CI 74%, 92%) and a retention rate of 90% (CI 81%, 99%). Completed follow-up on 28 participants will allow the Roland Morris Disability mean score to be estimated, with an approximate standard error of 0.98, assuming a SD of 6, thus enabling future power calculations to be calculated. Sample sizes between 24 and 40 are recommended for a feasibility study [38-40] and thus by recruiting 30 participants, we are confident we can determine the feasibility of conducting a definitive trial.

Data Analysis

A statistical analysis plan will be finalised and approved prior to database lock and commencement of data analysis. Feasibility outcomes will be summarised using appropriate descriptive statistics; mean (95% Confidence Interval) for continuous and frequency (%) for categorical. Completeness and descriptive summaries of outcome data at each follow up time point will be presented. Descriptive summaries of NHS costs (using standard unit costs) data at each follow up time point will be presented.

Economic Evaluation

In addition, at week 8, to inform the definitive economic analysis, we will assess resource use between VP and MBNB treatments; the ease of access to information about resource use from routine database systems; and the feasibility of collecting such data.

Surgical VP and MBNB treatment resource use will be collected from the medical notes and through discussions with participants by the research staff. The cost associated with the surgery will be based on the recorded resource use for the surgery (eg. Consumables, equipment, grade and number of nursing staff present during the operation). Further health resource information will be extracted from the hospital electronic system (NotIS) and the GP electronic systems by the research assistant. This will include any outpatient appointment, outpatient procedures, emergency department visits, inpatient admissions related to the study or GP visits captured at baseline, week 4 and week 8.

The unit costs of health resources will be based on national tariffs such as the Unit Costs of Health and Social Care for primary care resources, NHS Reference Costs for secondary care resources and the BNF for prescriptions. These tariffs will be estimated at 2021 prices. We will also utilise national databases such as the NHS Healthcare Resource Group (HRG) Tariff [41], the Personal Social Services Research Unit (PSSRU) Costs of Health [42], the Office of National Statistics (ONS) Bulletin Annual Survey of Hours and Earnings[43] and NHS Reference

For peer review only

Costs (https://www.gov.uk/government/collections/nhs-reference-costs). Any unit cost that is not available will be estimated in consultation with the hospital finance department.

Health Economic Analysis

We will rehearse the cost-effective analysis to inform the study hypothesis and the analysis plan for the definitive trial. The within-trial economic evaluation will determine the cost and outcome of VP and MBNB treatment from a NHS perspective. The evaluation will follow the reference case guidance for technology appraisals as set out by NICE[44] .Effectiveness will be captured using quality adjusted life years (QALYs) as assessed by the EQ-5D-5L[31] at 8 weeks. The primary health economic outcome of the evaluation will be the incremental cost per additional quality-adjusted life years (QALY) gained from surgical VP and MBNB. To control for the impact of uncertainty, one- and two-way sensitivity analyses will be performed on (but not exclusively) age, gender and baseline scores. The impact of parameter uncertainty will also be addressed using a probabilistic sensitivity analysis, allowing the calculation of 95% confidence intervals for the incremental cost-effectiveness ratio and cost-effectiveness acceptability curves.

Qualitative analysis

Data will handled using the NVivo software package and analysed using a framework thematic approach. The framework will be informed by the literature around the challenges of clinical trial methodology[45-49], and initial themes are likely to include elements such as randomisation, outcome measures, communication and feedback. Data from each interview will be mapped to the thematic tables.

Harms

The intervention is not testing a new surgical technique or medical treatment, and VP and MBNB are recognised as safe and routinely used procedures, hence AEs will be recorded in the CRFs, but will not require expedited reporting to the sponsor or REC.

All AEs will be reviewed by the chief investigator (CI) and recorded as part of the study outcome measures with an assessment of the severity, relation and expectation. All deaths occurring up to the final study visit and serious AEs, other than expected surgical complications, will be recorded on the Sponsor SAE form and emailed to the Sponsor within 3 days of a researcher becoming aware of the event. Those related to the study and unexpected will be reported to the REC within 15 days. Events will be followed up until resolved or a final outcome has been reached.

ETHICS and DISSEMNATION

Patient and Public involvement

The study's Patient and Public Involvement (PPI) group are members of the Royal Osteoporosis Society's Nottingham Support group. The topic is well recognized amongst the PPI who highlighted this area for further research. The member's experiences have greatly influenced the design of the research study and the choice of proposed outcome measures of

For peer review only

the study. They will be able to comment on aspects of the study design and contribute to production of questionnaire booklets, information sheets and other documents to ensure these are understandable and acceptable to patients. They will also be invited to comment on methods of sharing the study findings and support the writing of the definitive, future trial application.

Dissemination Policy

The team's dissemination strategy aims to target relevant policy makers and patient groups through our PPI representative. We will publish the study protocol and the full report of AVERT will be available on the NIHR RfPB website. Results from the trial will also be submitted for presentation at scientific meetings and conferences targeted at clinicians working with older people, trauma and spinal surgery.

Study Registration and Approvals

AVERT has received approval from the Research Ethics Committee (REC—Yorkshire & The Humber – Bradford Leeds, reference number 21/YH/0065), Health Research Authority (HRA) and the Nottingham Queens Medical Centre Research & Innovation department. Nottingham University Hospitals NHS Trust will act as sponsor to this study. The study has been registered on a clinical trials database (https://www.isrctn.com, reference number ISRCTN 18334053, pre-results).

DISCUSSION

Across Europe, 22 million women and 5 million men have osteoporosis, with 4 million new fragility fractures occurring a year, at an estimated health and social care cost of £37 billion [50] which is predicted to double by 2050 [51]. One in 2 women and 1 in 5 men over the age of 50 will experience and osteoporotic fracture in their lifetime, with this rising by 25% over the next 5 years [52]. In the UK, around 3.5 million people have osteoporosis, with an annual incidence of 500,000 of new fragility fractures [53]. Vertebral fragility fractures (VFF) are the most common osteoporotic fracture. Prevalence studies suggest 20% of women aged over 80 years have sustained a VFF [54].

The majority of those with pain symptoms will have mild to moderate pain, however a significant proportion have severe pain and require admission to hospital. The annual incidence of hospitalisation with symptomatic VFF is rising (50 per 10,000/year in those aged 80 years and over) [5], and these figures could be still be underestimated as up to 70% of vertebral fractures remain undiagnosed [5, 55]. In one study, it was reported that 34% of patients with VFF required acute hospital admission [56], with an average length of stay of 15 days in the UK [57]. Hospitalised VFF patients tend to be older, frail with coexisting comorbidities, cognitive impairment and have worse physical-health-related quality of life [58]. With the increasing numbers of VFF and more patients requiring acute hospitalisation, the healthcare resource burden within this group of patients is alarming.

Current NICE guidance on management of VFF emphasises a trial of pain optimisation before surgical intervention (vertebral augmentation) is considered. Potent morphine based analgesia, which are frequently required, lead to nausea, constipation and particularly in those with cognitive impairment, delirium [11]. Vertebral augmentation is a general term for several

For peer review only

techniques used to treat painful VFF, with the aim of consolidating the fracture and, when possible, achieve height restoration. Vertebroplasty (VP) is a minimally invasive, image-guided key-hole procedure that involves injection of radio-opaque bone cement into the fractured vertebral body, in an effort to provide pain relief and stability [13]. Percutaneous balloon kyphoplasty (PBK) attempts to restore vertebral body height by inflating a balloon prior to bone cement injection [59]. Although the recent Cochrane review has debated the effectiveness of VP [15, 16, 17], limitations in the review are recognised [16, 17]. Emerging evidence suggests that the optimal benefits are seen when VP is performed within 3 weeks of fracture in hospitalised older patients [19, 20]

Debate has been generated regarding the effectiveness of VP and its application in patients with vertebral fractures following a Cochrane review. However, the data is limited for VP and its application for inpatients [18, 19], even less so for older hospitalized patients. The VAPOUR trial, which had a subgroup of inpatients (59%) with vertebral fractures, demonstrated that vertebroplasty showed benefit in terms of pain reduction mortality, length of stay in hospital [19]. The other trials (VERTOS4 [60], Kalllmes et al [61], Buchbinder et al [62]) highlighted in the Cochrane review had no inpatients and recruited from an outpatient setting. As far as we are aware, the AVERT trial will be the first trial to focus exclusively on hospitalised older people.

It is also worth noting that previous trials did not include patients who had a diagnosis of dementia or delirium during recruitment. As cognitively impaired older patients presenting to hospital with VFF account for a large proportion of the real world VFF cohort [63], it would severely affect the validity and generalisability of the trial to exclude them. However, we are unaware of any published RCT on VP that have included patients with cognitive impairment. We believe that this cohort of patients would benefit most from early intervention for their painful VFF as they may struggle to comply with regular analgesia intake, and opiate based analgesia which is usually required may have subsequent side effects that will precipitate delirium itself.

Additionally, our participants will receive treatment for their painful VFF with either VP, a NICE approved treatment for VFF, as our control group, or our trial intervention with MBNB. Should MBNB prove as effective as VP in controlling pain, it also negates the need for a general anaesthetic for this population group, which is viewed as potentially more harmful in the frail, older population. In this trial, we also propose that surgical management should be considered earlier in the treatment of hospitalisation , improving pain with the aim of preventing pain-related immobilisation and disability, therefore reducing risks of opiate induced side effects and consequences of prolonged immobilisation. The timely intervention (within 72 hours of randomisation) we propose in this feasibility trial will be dependent on other factors such as timely identification of acute VFF, MRI imaging availability, operator and theatre availability, where more emergent procedures will take precedence. This is in addition to the already growing emergency list from the impact of the COVID-19 pandemic [64, 65.]

The alternative solution to this is to devise a new clinical pathway for these patients. Hence it is essential that the feasibility trial is delivered within the framework of the existing healthcare service in order to facilitate implementation. As we may be potentially expanding clinical workload in other departments (ie interventional radiology and anaesthesia) should MBNB prove effective, the feasibility of this must be explored and limitations and constraints in an already pressured healthcare system. Cost effectiveness of MBNB must also be explored compared to standard care VP, hence the inclusion of an economic evaluation. A benefit of a

For peer review only - http://bmjopen.bmj.com/site/about/guidelines.xhtml

new clinical service would be that MBNB could be delivered in local hospitals via anaesthetic or interventional radiology departments. This facilitates early treatment for painful VFF and eliminates the need for our patients to travel to regional spine centres for VP. This sentiment was echoed in a UK study which highlighted concerns regarding access to VP nationally for our older hospitalised patients with VFF [66].

The key outcomes address questions posed by the possible limitations of conducting such a trial within an existing public health service, specifically to recruitment and adherence to randomisation. We anticipate that this feasibility study will provide information and data to test our hypothesis that MBNB is as cost effective and safe compared to VP for the treatment of acute VFF in hospitalised older patients. With national organisational leads pushing for better outcomes for frail older patients [67, 68], we hope that this trial will be a further catalyst for change nationally in the management of older hospitalised patients with VFF.

Trial Status

The trial is currently open for recruitment, with 12 patients currently enrolled (November 2021). We aim to complete the study in September 2022.

Author affiliations:

1. Healthcare of the Older People Division, Nottingham University Hospitals NHS Trust, Nottingham, UK
2. Department of Radiology, Nottingham University Hospitals NHS Trust, Nottingham, UK
3. Division of Epidemiology & Public Health, University of Nottingham, Nottingham UK
4. Nottingham University Business School, University of Nottingham, Nottingham, UK
5. Department of Anaesthesia, Nottingham University Hospitals NHS Trust, Nottingham, UK
6. School of Health Sciences, University of Nottingham, Nottingham, UK
7. Derby Clinical Trials Support Unit, University Hospitals of Derby and Burton NHS Foundation Trust
8. Centre for Spinal Studies and Surgery, Nottingham University Hospitals NHS Trust, Nottingham, UK
9. Division of Rehabilitation, Ageing and Wellbeing, University of Nottingham, Nottingham, UK
10. National Institute for Health Research (NIHR) Nottingham Biomedical Research Centre (BRC), Nottingham, UK

Acknowledgements

We would like to express our sincere thanks and gratitude to our PPI representative (**LW**) for her contribution to the original protocol and the NIHR RfPB for funding this study.

Author Contributors

For peer review only

CWT wrote the manuscript. **YA, AB, SB, MC, AD, DP, LW and OS** are all key protocol contributors providing their expertise on specific aspects of the study. **OS** is the chief investigator of the study. **MC** is the trial co-ordinator. **AF** is the lead trial statistician. All authors have read, contributed amendments to and approved the final manuscript.

Funding

This paper presents independent research funded by the National Institute for Health Research (NIHR) under its Research for Patient Benefit (RfPB) Programme. (Grant Reference number **NIHR RfPB-201937**)

Disclaimer

Views and opinions expressed in this publication are those of the authors.

Neither sponsor nor funder had or will have direct input in the study conduct, data analysis, data interpretation, and manuscript writing and dissemination findings.

Competing Interests

None Declared.

Patient consent for publication.

Not Required.

Provenance and peer review

Not commissioned; peer-reviewed for ethical and funding approval prior to submission.

For peer review only

REFERENCES

1. Silverman SL, Minshall ME, Shen W, et al., Health-Related Quality of Life Subgroup of the Multiple Outcomes of Raloxifene Evaluation Study. The relationship of health-related quality of life to prevalent and incident vertebral fractures in postmenopausal women with osteoporosis: results from the Multiple Outcomes of Raloxifene Evaluation Study. Arthritis Rheum 2001;44:2611–19.
2. Cohen LD. Fractures of the osteoporotic spine. Orthop Clin N Am 1990;21:143–50.
3. Melton LJ 3rd, Atkinson EJ, Cooper C, O'Fallon WM,Riggs BL. Vertebral fractures predict subsequent fractures. Osteoporosis Int. 1999;10:214-21.
4. Black DM, Arden NK, Palermo L, Pearson J, Cummings SR. Prevalent vertebral deformities predict hip fractures and new vertebral deformities but not wrist fractures. Study of Osteoporotic Fractures Research Group. J Bone Miner Res 1999;14:821-28.
5. Cooper C1, Atkinson EJ, Jacobsen SJ, O'Fallon WM, Melton LJ 3rd. Population-based study of survival after osteoporotic fractures. Am J Epidemiol 1993 May;137:1001-5.
6. Jacobsen SJ, Cooper C, Gottlieb MS, Goldberg J, Yahnke DP, Melton LJ. Hospitalization with vertebral fracture among the aged: a national population-based study, 1986–1989. Epidemiology 1992; 3: 515–18.
7. Ong T, Kantachuvesiri P, Sahota O, Gladman JRF. Characteristics and outcomes of hospitalised patients with vertebral fragility fractures: a systematic review. Age Ageing. 2018 Jan 1;47(1):17-25.
8. Walters S, Chan S, Goh L, Ong T, Sahota O. The Prevalence of Frailty in Patients Admitted to Hospital with Vertebral Fragility Fractures. Curr Rheumatol Rev 2016;12:244-247.
9. Abe T, Shibao Y, Takeuchi Y, Mataki Y, Amano K, Hioki S, Miura K, Noguchi H, Funayama T, Koda M, Yamazaki M. Initial hospitalization with rigorous bed rest followed by bracing and rehabilitation as an option of conservative treatment for osteoporotic vertebral fractures in elderly patients: a pilot one arm safety and feasibility study. Arch Osteoporos 2018; 23:134-138.
10. Goldstein CL, Chutkan NB, Choma TJ, Orr RD. Management of the elderly with vertebral compression fractures. Neurosurgery 2015; 77:33–45.
11. Chau DL, Walker V, Pai L, Cho LM. Opiates and elderly: Use and side effects Clin Interv Aging 2008; 3:273–278.
12. Tsai YW, Hsiao FY, Wen YW, et al. Clinical outcomes of vertebroplasty or kyphoplasty for patients with vertebral compression fractures: a nationwide cohort study. J Am Med Dir 2013;14:41–47
13. Lieberman IH, Togawa D, Kayanja MM. Vertebroplasty and kyphoplasty: filler materials. Spine J. 2005;5:S305–16.
14. Tsoumakidou G, CW, Koch G, et al. CIRSE Guidelines on Percutaneous Vertebral Augmentation. Cardiovasc Intervent Radiol 2017; 40:331–342.
15. Cochrane Database of Systematic Reviews. Percutaneous vertebroplasty for osteoporotic vertebral compression fracture. Cochrane Systematic Review - Intervention Version published: 06 November 2018; https://doi.org/10.1002/14651858.CD006349.pub4.
16. Clark W, Bird P, Diamond T et al. EBM Analysis General medicine, Cochrane vertebroplasty review misrepresented evidence for vertebroplasty with early intervention in severely affected patients http://dx.doi.org/10.1136/bmjebm-2019-11117.
17. https://www.cochranelibrary.com/cca/doi/10.1002/cca.2376/full .
18. Diamond T, Clark W, Bird P et al. Early vertebroplasty within 3 weeks of fracture for acute painful vertebral osteoporotic fractures: subgroup analysis of the VAPOUR trial and review of the literature.
European Spine Journal. 2020: https://doi.org/10.1007/s00586-020-06362-2.
19. Corteta B, Chastanet P, Laredo J-D. Plea for continuing but rational use of vertebroplasty for osteoporotic vertebral fractures. Joint Bone Spine 2020;87:281–283.

20. Zhu RS, Kan SL, Ning GZ, Chen LX, Cao ZG, Jiang ZH, Zhang XL, Hu W. Which is the best treatment of osteoporotic vertebral compression fractures: balloon kyphoplasty, percutaneous vertebroplasty, or non-surgical treatment? A Bayesian network meta-analysis. Osteoporos Int 2019;30:287-298.
21. Zhang L, Zhai P. A Comparison of Percutaneous Vertebroplasty Versus Conservative Treatment in Terms of Treatment Effect for Osteoporotic Vertebral Compression Fractures: A Meta-Analysis. Surg Innov 2020;27:19-25.
22. Maravic M, Taupin P, Roux C. Hospital burden of vertebral fractures in France: influence of vertebroplasty. Osteoporosis Int 2013;24:2001–06.
23. Edidin AA, Ong KL, Lau E, et al. Morbidity and mortality after vertebral fractures: comparison of vertebral augmentation and nonoperative management in the Medicare population. Spine 2015;40:1228–41.
24. Lange A, Kasperk C, Alvares L, et al. Survival and cost comparison of kyphoplasty and percutaneous vertebroplasty using German claims data. Spine 2014;39:318–26.
25. Lin JH, Chien LN, Tsai WL, et al. Early vertebroplasty associated with a lower risk of mortality and respiratory failure in aged patients with painful vertebral compression fractures: a population-based cohort study in Taiwan. Spine J 2017;17:1310–18.
26. Saracen A, Kotwica Z. Complications of percutaneous vertebroplasty: an analysis of 1100 procedures performed in 616 patients. Medicine (Baltimore). 2016;95:e3850.
27. Mitra R, Do H, Alamin T, Cheng I. Facet pain in thoracic compression fractures. Pain Med 2010;11:1674-1677
28. Bae IS, Chun HJ, Bak KH, Yi HJ, Choi KS, Kim KD. Medial Branch Block Versus Vertebroplasty for 1- Level Osteoporotic Vertebral Compression Fracture: 2-Year Retrospective Study. World Neurosurg. 2019 Feb;122:e1599-e1605.
29. Roland MO, Morris RW. A study of the natural history of back pain. Part 1: Development of a reliable and sensitive measure of disability in low back pain. Spine 1983; 8: 141-144.
30. McCaffery M, Pasero C. Pain: Clinical Manual, St. Louis, 1999, P. 16. Copyrighted by Mosby, Inc.
31. http://www.euroqol.org/eq-5d-products/eq-5d-5l.html.
32. Green J and Young J. A test-retest reliability study of the Barthel Index, the Rivermead Mobility Index, the Nottingham extended Activities of Daily Living Scale and the Frenchay Activities Index. Journal Disability and Rehabilitation 2001; 23: 234-39.
33. Hodkinson HM. Evaluation of a mental test score for assessment of mental impairment in the elderly. Age Ageing. 1972;1(4):233-8.
34. Nasreddine ZS, Phillips NA, Bédirian V, et al. The Montreal Cognitive Assessment, MoCA: a brief screening tool for mild cognitive impairment. J Am Geriatr Soc. 2005;53(4):695–9.
35. Charlson ME, Pompei P, Ales KL, MacKenzie CR. A new method of classifying prognostic comorbidity in longitudinal studies: development and validation. J Chronic Dis 1987;40(5):373-383.
36. Rockwood K, Song X, MacKnight C, et al. A global clinical measure of fitness and frailty in elderly people. CMAJ. 2005;173(5):489–95.
37. Faculty of Pain Medicine. Opioid Dose Equivalence: calculation of oral Morphine Equivalent Daily Dose (oMEDD). Australian and New Zealand College of Anaesthetists. Available at: http://www.fpm.anzca.edu.au/resources/professional-documents/ OPIOID%20DOSE%20EQUIVALENCE.pdf. Date accessed March 2015.
38. Watson J, Torgerson D. Increasing recruitment to randomised trials: a review of randomised controlled trials. BMC Med Res Methodol 2006; 6: 34-38.
39. Treweek S, Pitkethly M, Kjeldstrom M, Taskila T, Johansen M, Sullivan F et al. Strategies to improve

For peer review only

recruitment to randomised clinical trials (Review). Cochrane Database SystRev 2010; Issue 4: No. MR000013.
40. Browne RH. On the use of a pilot sample for sample size determination. Stat Med. 1995; 14:1933–1940.
41. https://improvement.nhs.uk/resources/national-tariff-2021-consultation/
42. https://www.pssru.ac.uk/project-pages/unit-costs/unit-costs-2019/
43. https://www.ons.gov.uk/surveys/informationforbusinesses/businesssurveys/annualsurveyofhoursandearningsashe
44. National Institute for Health and Care Excellence. NICE Technology Appraisal Guidance. https://www. nice. org. uk/ about/ what- we- do/ our- programmes/ nice- guidance/ nice- technology- appraisal- guidance (Accessed May 2019).
45. Watson J, Torgerson D. Increasing recruitment to randomised trials: a review of randomised controlled trials. BMC Med Res Methodol 2006; 6: 34-38.
46. Treweek S, Pitkethly M, Kjeldstrom M, Taskila T, Johansen M, Sullivan F et al. Strategies to improve recruitment to randomised clinical trials (Review). Cochrane Database SystRev 2010; Issue 4: No. MR000013.
47. Browne RH. On the use of a pilot sample for sample size determination. Stat Med. 1995; 14:1933–1940.
48. Lancaster GA, Dodd S, Williamson PR. Design and analysis of pilot studies: recommendations for good practice. J Eval Clin Practice 2004;10:307-312
49. Sim J, Lewis M. The size of a pilot study for a clinical trial should be calculated in relation to considerations of precision and efficiency. J Clin Epidemiol 2012;65:301-308.
50. Hernlund E, Svedbom A, Ivergård M, Compston J, Cooper C, Stenmark J, McCloskey EV, Jönsson B, Kanis JA. Osteoporosis in the European Union: medical management, epidemiology and economic burden. A report prepared in collaboration with the International Osteoporosis Foundation (IOF) and the European Federation of Pharmaceutical Industry Associations (EFPIA). Arch Osteoporos 2013;8:136-142.
51. Reginster JY, Burlet N. Osteoporosis: A still increasing prevalence. Bone. 2006 Feb;38 (2 Suppl 1):S4-9.
52. Borgström F, Zethraeus N, Johnell O, et al. Costs and quality of life associated with osteoporosis related fractures in Sweden. Osteoporos Int 2006;17:637–50.
53. BROKEN BONES, BROKEN LIVES: A roadmap to solve the fragility fracture crisis in the United Kingdom.
54. O'Neill TW, Felsenberg D, Varlow J, Cooper C, Kanis JA, Silman J. The prevalence of vertebral deformity in European men and women: the European Vertebral Osteoporosis Study. J Bone Miner Res. 1996;11:1010-18.
55. Alendronate, etidronate, risedronate, raloxifene, strontium ranelate and teriparatide for the secondary prevention of osteoporotic fragility fractures in postmenopausal women (TA161). NICE: 2008. Available from: https://www.nice. org.uk/guidance/ta161 [Last updated 9 Aug. 2017].
56. Johansen A et al. Trauma admissions in the elderly how does a patient's age affect the likelihood of their being admitted to hospital after a fracture? Injury Vol 29, No 10.
57. Osteoporosis in the European Union 2008. Ten years of Progress and ongoing challenges. International Osteoporosis foundation.
58. Al-Sari U, Tobias J, Clark E. Health related quality of life in older people with osteoporotic vertebral fractures: a systematic review and meta-analysis. Osteoporosis Int. 2016;27(10),2891-909.
59. Robinson Y, Heyde CE, Fo¨rsth P, Olerud C. Kyphoplasty in osteoporotic vertebral compression fractures—guidelines and technical considerations. J Orthop Surg Res 2011;6:43-47.
60. Firanescu C, Lohle PN, de Vries J, et al. VERTOS IV study group. Arandomised sham controlled trial of vertebroplasty for painful acute osteoporotic vertebral fractures (VERTOS IV). Trials 1745;2011:12–93.

For peer review only

61. Kallmes DF, Comstock BA, Heagerty PJ, et al. A randomized trial of vertebroplasty for osteoporotic spinal fractures. New England Journal of Medicine 2009;361:569–79.
62. Buchbinder R, Osborne RH, Ebeling PR, et al. A randomized trial of vertebroplasty for painful osteoporotic vertebral fractures. N Engl J Med2009;361:557–68.
63. Ong T, Sahota O, Gladman JRF. The Nottingham Spinal Health (NoSH) Study: a cohort study of patients hospitalised with vertebral fragility fractures. Osteoporosis International 2020; 31:363–370
64. Too long to wait: the impact of COVID-19 on elective surgery Lancet Rheumatology, ISSN: 2665-9913, Vol: 3, Issue: 2, Page: e83; 2021.
65. Covid-19: Waiting times in England reach record highsBMJ 2020; 370 doi: https://doi.org/10.1136/bmj.m3557
66. Ong T, Gladman JRF, Sahota O. Management of Patients Admitted of Hospital with Vertebral Fractures-A Modified Delphi Study. Age and Ageing. 2020: In press
67. https://www.scfn.org.uk/SpecialisedClinicalFrailtyToolkitVersion4 - December 2019
68. https://www.gettingitrightfirsttime.co.uk/spinal-surgery-report/

For peer review only

Figure 1.
Legend/Caption: Flowchart illustrating consent process for the AVERT trial

Figure 2
Legend/Caption: Participant flow through the trial including the timings of data collection

For peer review only

Figure 1. Flowchart Guidance for Consent

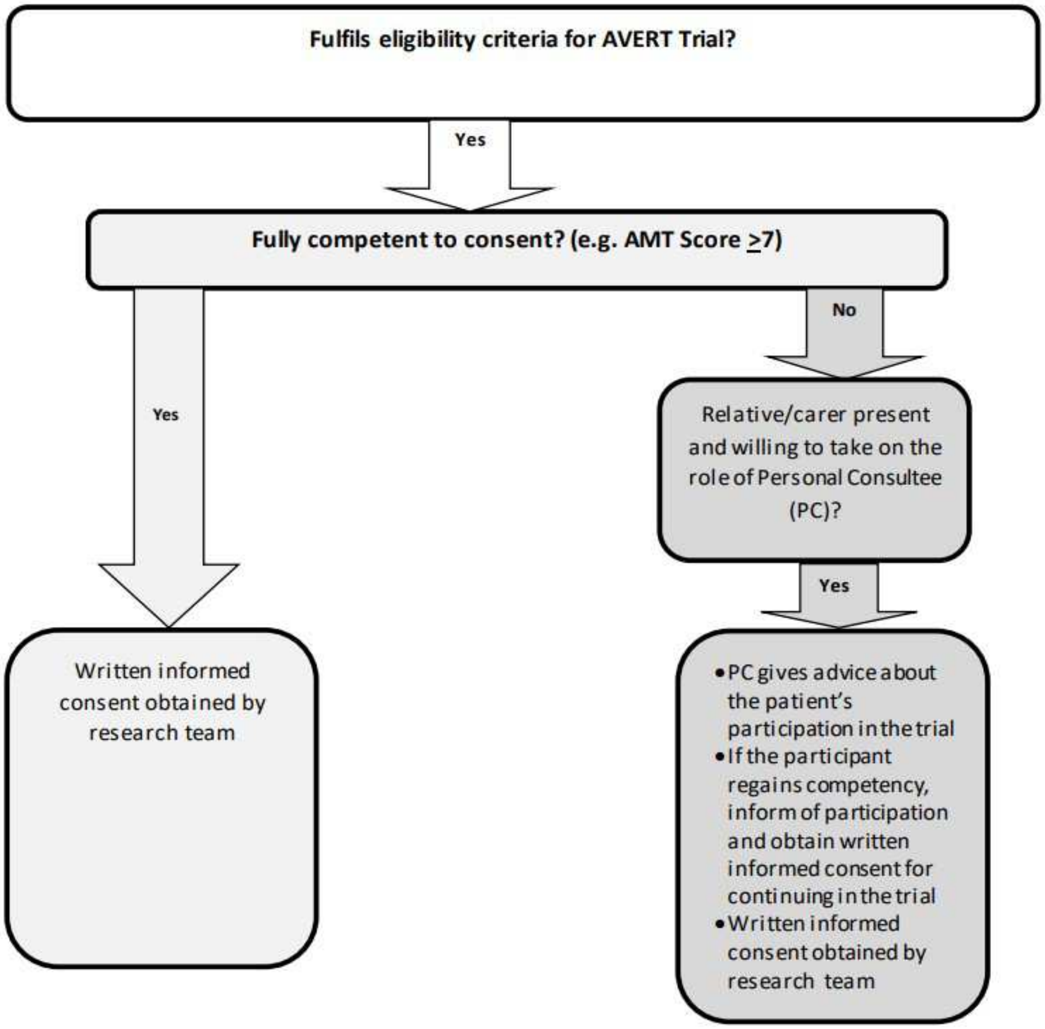

If a participant loses capacity during the study period, they will continue in the study unless there is a known change in their views/perspectives determined by liaising with their relative/carer.

For participants who initially lack capacity but later regain capacity, eg with delirium we will re-approach them, detailing their participation in the study to date, provide a patient information sheet and discuss the purpose and nature of the research, its benefits (or lack of), risks and burdens; understand the alternatives to taking part. If they agree to continue with participation in the study, we will request to sign and updated consent form. If they decline they will be categorised as withdrawn.

Figure 2. TRIAL FLOW CHART

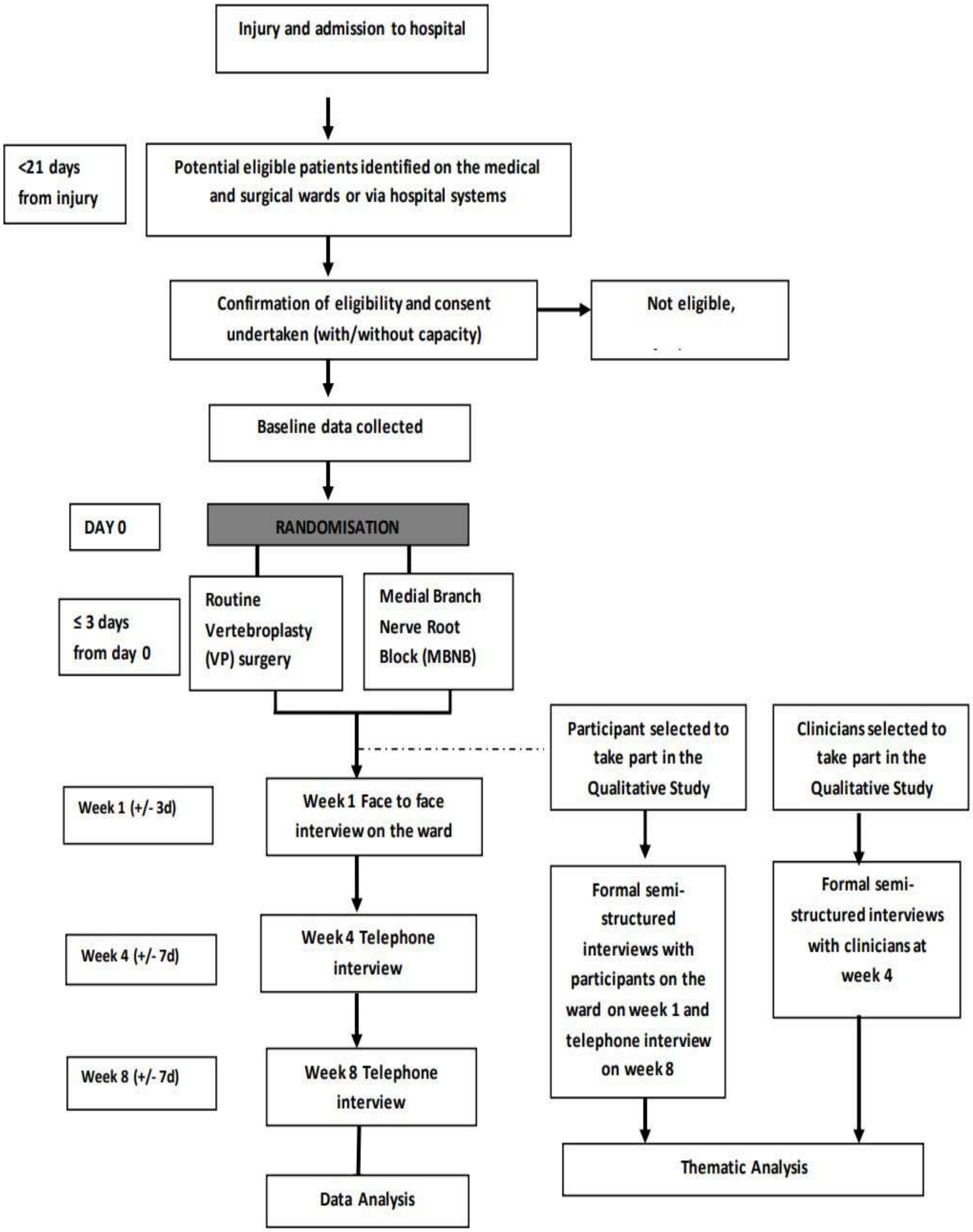

AVERT
Acute VertEbRal augmentaTion study

Nottingham University Hospitals NHS
NHS Trust

Participant Consent Form
(Patient)

The AVERT (Acute Vertebral Fracture Augmentation) Study

Chief Investigator: Professor Opinder Sahota

Patient Screening No.: **SN** ☐☐☐ Initials: ☐☐☐

Patient initial each box

1. I confirm that I have read and understood the information sheet dated (/ /) (Version ___ for the above study and have had the opportunity to ask questions. ☐
2. I understand that my participation is voluntary and that I am free to withdraw at any time without my medical care or legal rights being affected. ☐
3. I understand that I may be invited to take part in a qualitative interview and that written quotations from the interview, that will be anonymized, may be used in publications, reports and presentations ☐
4. I understand that my medical records and any other information collected for the study may be looked at by authorised individuals from the Sponsor for the study, the funding body, and the UK Regulatory Authority in order to check that the study is being carried out correctly. ☐
5. I understand that even if I withdraw or am withdrawn from the above named study, that the data already collected from me will be retained and used when analysing the results of the study. ☐
6. I consent to the secure storage including electronic, of personal information for the purposes of this study. I understand that any information that could identify me will be kept strictly confidential and that no personal information will be included in the study report, presentations or other publication. ☐
7. I agree to undergo, where required, the tests and procedures as described in the information sheet. ☐
8. I agree that my GP, or any other doctor treating me, will be notified of my participation in this study. ☐
9. I agree to take part in the study ☐

Name of the patient *(Print)*	date	Patient's signature
Name of person taking consent *(Print)*	date	Signature

Original to be retained and filed in the site file. 1 copy to patient, 1 copy to be filed in patient's notes.

For peer review only

For peer review only - http://bmjopen.bmj.com/site/about/guidelines.xhtml

SPIRIT 2013 Checklist: Recommended items to address in a clinical trial protocol and related documents*

Section/item	Item No	Description
**Administrative information**		
Title (Page 1)	1	Descriptive title identifying the study design, population, interventions, and, if applicable, trial acronym
Trial registration (Page 2 & 11)	2a	Trial identifier and registry name. If not yet registered, name of intended registry
	2b	All items from the World Health Organization Trial Registration Data Set
Protocol version Not applicable	3	Date and version identifier
Funding (Page 14)	4	Sources and types of financial, material, and other support
Roles and responsibilities (Page 1& 14)	5a	Names, affiliations, and roles of protocol contributors
	5b	Name and contact information for the trial sponsor
Not applicable	5c	Role of study sponsor and funders, if any, in study design; collection, management, analysis, and interpretation of data; writing of the report; and the decision to submit the report for publication, including whether they will have ultimate authority over any of these activities
Not applicable	5d	Composition, roles, and responsibilities of the coordinating centre, steering committee, endpoint adjudication committee, data management team, and other individuals or groups overseeing the trial, if applicable (see Item 21a for data monitoring committee)
**Introduction**		
Background and rationale (Page 3)	6a	Description of research question and justification for undertaking the trial, including summary of relevant studies (published and unpublished) examining benefits and harms for each intervention
(Page 4)	6b	Explanation for choice of comparators
Objectives (Page 3)	7	Specific objectives or hypotheses

For peer review only

Trial design (Page 5)	8	Description of trial design including type of trial (eg, parallel group, crossover, factorial, single group), allocation ratio, and framework (eg, superiority, equivalence, noninferiority, exploratory)

Methods: Participants, interventions, and outcomes

Study setting (Page 5)	9	Description of study settings (eg, community clinic, academic hospital) and list of countries where data will be collected. Reference to where list of study sites can be obtained
Eligibility criteria (Page 5)	10	Inclusion and exclusion criteria for participants. If applicable, eligibility criteria for study centres and individuals who will perform the interventions (eg, surgeons, psychotherapists)
Interventions (Page 6 & 7)	11a	Interventions for each group with sufficient detail to allow replication, including how and when they will be administered
	11b	Criteria for discontinuing or modifying allocated interventions for a given trial participant (eg, drug dose change in response to harms, participant request, or improving/worsening disease)
	11c	Strategies to improve adherence to intervention protocols, and any procedures for monitoring adherence (eg, drug tablet return, laboratory tests)
	11d	Relevant concomitant care and interventions that are permitted or prohibited during the trial
Outcomes (Page 7)	12	Primary, secondary, and other outcomes, including the specific measurement variable (eg, systolic blood pressure), analysis metric (eg, change from baseline, final value, time to event), method of aggregation (eg, median, proportion), and time point for each outcome. Explanation of the clinical relevance of chosen efficacy and harm outcomes is strongly recommended
Participant timeline (Page 8)	13	Time schedule of enrolment, interventions (including any run-ins and washouts), assessments, and visits for participants. A schematic diagram is highly recommended (see Figure)
Sample size (Page 9)	14	Estimated number of participants needed to achieve study objectives and how it was determined, including clinical and statistical assumptions supporting any sample size calculations
Recruitment (Page 6)	15	Strategies for achieving adequate participant enrolment to reach target sample size

Methods: Assignment of interventions (for controlled trials)

Allocation:

Sequence generation (Page 6)	16a	Method of generating the allocation sequence (eg, computer-generated random numbers), and list of any factors for stratification. To reduce predictability of a random sequence, details of any planned restriction (eg, blocking) should be provided in a separate document that is unavailable to those who enrol participants or assign interventions
Allocation concealment mechanism (Page 6)	16b	Mechanism of implementing the allocation sequence (eg, central telephone; sequentially numbered, opaque, sealed envelopes), describing any steps to conceal the sequence until interventions are assigned
Implementation (Page 6)	16c	Who will generate the allocation sequence, who will enrol participants, and who will assign participants to interventions
Blinding (masking) (Page 6)	17a	Who will be blinded after assignment to interventions (eg, trial participants, care providers, outcome assessors, data analysts), and how
(not applicable)	17b	If blinded, circumstances under which unblinding is permissible, and procedure for revealing a participant's allocated intervention during the trial
**Methods: Data collection, management, and analysis**		
Data collection methods (Page 7-9)	18a	Plans for assessment and collection of outcome, baseline, and other trial data, including any related processes to promote data quality (eg, duplicate measurements, training of assessors) and a description of study instruments (eg, questionnaires, laboratory tests) along with their reliability and validity, if known. Reference to where data collection forms can be found, if not in the protocol
(not applicable)	18b	Plans to promote participant retention and complete follow-up, including list of any outcome data to be collected for participants who discontinue or deviate from intervention protocols
Data management Page 9	19	Plans for data entry, coding, security, and storage, including any related processes to promote data quality (eg, double data entry; range checks for data values). Reference to where details of data management procedures can be found, if not in the protocol
Statistical methods (Page 9)	20a	Statistical methods for analysing primary and secondary outcomes. Reference to where other details of the statistical analysis plan can be found, if not in the protocol
Not applicable	20b	Methods for any additional analyses (eg, subgroup and adjusted analyses)
Page 9 & 10	20c	Definition of analysis population relating to protocol non-adherence (eg, as randomised analysis), and any statistical methods to handle missing data (eg, multiple imputation)

Methods: Monitoring

Data monitoring (Page 9)	21a	Composition of data monitoring committee (DMC); summary of its role and reporting structure; statement of whether it is independent from the sponsor and competing interests; and reference to where further details about its charter can be found, if not in the protocol. Alternatively, an explanation of why a DMC is not needed
Not applicable	21b	Description of any interim analyses and stopping guidelines, including who will have access to these interim results and make the final decision to terminate the trial
Harms (Page 10)	22	Plans for collecting, assessing, reporting, and managing solicited and spontaneously reported adverse events and other unintended effects of trial interventions or trial conduct
Auditing N/A	23	Frequency and procedures for auditing trial conduct, if any, and whether the process will be independent from investigators and the sponsor

Ethics and dissemination

Research ethics approval (Page 11)	24	Plans for seeking research ethics committee/institutional review board (REC/IRB) approval
Protocol amendments (Page 11)	25	Plans for communicating important protocol modifications (eg, changes to eligibility criteria, outcomes, analyses) to relevant parties (eg, investigators, REC/IRBs, trial participants, trial registries, journals, regulators)
Consent or assent (Page 6)	26a	Who will obtain informed consent or assent from potential trial participants or authorised surrogates, and how (see Item 32)
Not applicable	26b	Additional consent provisions for collection and use of participant data and biological specimens in ancillary studies, if applicable
Confidentiality (6)	27	How personal information about potential and enrolled participants will be collected, shared, and maintained in order to protect confidentiality before, during, and after the trial
Declaration of interests (Page 14)	28	Financial and other competing interests for principal investigators for the overall trial and each study site
Access to data (Page 9)	29	Statement of who will have access to the final trial dataset, and disclosure of contractual agreements that limit such access for investigators
Ancillary and post-trial care (Page 10)	30	Provisions, if any, for ancillary and post-trial care, and for compensation to those who suffer harm from trial participation

For peer review only

Dissemination policy (Page 11)	31a	Plans for investigators and sponsor to communicate trial results to participants, healthcare professionals, the public, and other relevant groups (eg, via publication, reporting in results databases, or other data sharing arrangements), including any publication restrictions
	31b	Authorship eligibility guidelines and any intended use of professional writers
	31c	Plans, if any, for granting public access to the full protocol, participant-level dataset, and statistical code
**Appendices**		
Informed consent materials (Supplied as supplementary document)	32	Model consent form and other related documentation given to participants and authorised surrogates
Biological specimens (N/A)	33	Plans for collection, laboratory evaluation, and storage of biological specimens for genetic or molecular analysis in the current trial and for future use in ancillary studies, if applicable

*It is strongly recommended that this checklist be read in conjunction with the SPIRIT 2013 Explanation & Elaboration for important clarification on the items. Amendments to the protocol should be tracked and dated. The SPIRIT checklist is copyrighted by the SPIRIT Group under the Creative Commons "Attribution-NonCommercial-NoDerivs 3.0 Unported" license.

For peer review only
